# m^5^C modification of mRNA serves a DNA damage code to promote homologous recombination

**DOI:** 10.1038/s41467-020-16722-7

**Published:** 2020-06-05

**Authors:** Hao Chen, Haibo Yang, Xiaolan Zhu, Tribhuwan Yadav, Jian Ouyang, Samuel S. Truesdell, Jun Tan, Yumin Wang, Meihan Duan, Leizhen Wei, Lee Zou, Arthur S. Levine, Shobha Vasudevan, Li Lan

**Affiliations:** 1 Department of Microbiology and Molecular Genetics, University of Pittsburgh School of Medicine, UPMC Hillman Cancer Center, 5117 Centre Ave., Pittsburgh, PA 15213 USA; 2 Massachusetts General Hospital Cancer Center, Harvard Medical School, Boston, MA 02129 USA; 3 Department of Radiation Oncology, Massachusetts General Hospital, Harvard Medical School, Boston, MA 02129 USA; 4 Department of Pathology, Massachusetts General Hospital, Harvard Medical School, Boston, MA 02115 USA; 5 Department of Medicine, Massachusetts General Hospital, Harvard Medical School, Boston, MA 02129 USA

**Keywords:** Cancer epidemiology, Homologous recombination, DNA recombination, Epigenetics, RNA modification

## Abstract

Recruitment of DNA repair proteins to DNA damage sites is a critical step for DNA repair. Post-translational modifications of proteins at DNA damage sites serve as DNA damage codes to recruit specific DNA repair factors. Here, we show that mRNA is locally modified by m^5^C at sites of DNA damage. The RNA methyltransferase TRDMT1 is recruited to DNA damage sites to promote m^5^C induction. Loss of TRDMT1 compromises homologous recombination (HR) and increases cellular sensitivity to DNA double-strand breaks (DSBs). In the absence of TRDMT1, RAD51 and RAD52 fail to localize to sites of reactive oxygen species (ROS)-induced DNA damage. In vitro, RAD52 displays an increased affinity for DNA:RNA hybrids containing m^5^C-modified RNA. Loss of TRDMT1 in cancer cells confers sensitivity to PARP inhibitors in vitro and in vivo. These results reveal an unexpected TRDMT1-m^5^C axis that promotes HR, suggesting that post-transcriptional modifications of RNA can also serve as DNA damage codes to regulate DNA repair.

## Abstract (web summary)

Post-translational modifications of proteins at DNA damage sites can facilitate the recruitment of DNA repair factors. Here, the authors show that mRNA is locally modified with m^5^C at sites of DNA damage by the RNA methyltransferase TRDMT1 to promote homologous recombination repair.

## Introduction

Although the impact of transcription on DNA repair has been long appreciated, the functions of RNAs in DNA repair have just begun to emerge. Interestingly, although gene expression is locally suppressed by DNA damage, preexisting RNA transcripts and damage-induced non-coding RNAs at DNA damage sites may contribute to DNA repair^1–6^. Recent studies by others and us showed that DNA:RNA hybrids are induced by DNA double-stranded breaks (DSBs) and reactive oxygen species (ROS) in transcriptionally active regions of the genome^3, 7–9^. Furthermore, the DNA:RNA hybrids at sites of DNA damage promote recruitment of specific DNA repair proteins, thereby enhancing the efficiency and fidelity of DSB repair^9–11^. Notably, the function of RNA in recruiting DNA repair proteins is reminiscent to that of the chromatin flanking DSBs. A large body of literature has shown that the post-translational modifications (PTMs) of chromatin at DNA damage sites have a critical role in recruiting DNA repair proteins^12–14^. Many DNA repair proteins are in fact writers, readers, and erasers of various protein PTMs at DNA damage sites, highlighting the importance of DNA damage codes for DNA repair^15^. The similar functions of chromatin and RNA in the recruitment of DNA repair proteins raise an intriguing question as to whether RNA is also modified at sites of DSBs and whether the writers, readers, and erasers of RNA modifications are important for DSB repair.

To investigate whether RNA modifications are induced by DNA damage, we targeted the ROS-releasing protein KillerRed (KR) to a specific locus in the genome. Light activation of KR allows us to induce ROS and DNA damage locally at this site in either transcriptionally active or inactive state. Using this system, we found that the RNA modification m^5^C is specifically induced at the site of DNA damage in transcription-dependent manner. Furthermore, we found that TRDMT1, an RNA methyltransferase known to methylate tRNA^16–18^, is recruited to DNA damage sites and required for the induction of RNA m^5^C. Importantly, cells lacking TRDMT1 are defective for homologous recombination (HR), a pathway critical for DSB repair. These results reveal that HR is regulated by RNA m^5^C, a post-transcriptional DNA damage code on the mRNA at DSBs, and TRDMT1, the writer of this code.

We also pursued how the m^5^C DNA damage code is read by repair proteins at DNA damage sites. Loss of TRDMT1 impairs the localization of RAD51 and RAD52 to ROS-induced DNA damage. RAD52, which acts upstream of RAD51 in this context, displays a higher affinity for DNA:RNA hybrids containing m^5^C-modified RNA than hybrids without the modification, suggesting that RAD52 is one of the readers of m^5^C in HR. These results establish an unexpected, RNA modification-orchestrated TRDMT1–m^5^C–RAD52–RAD51 axis that promotes HR at ROS-induced DSBs.

In addition to its role in the HR at ROS-induced DSBs, TRDMT1 is also important for the canonical HR pathway operating at nuclease-generated DSBs. Consistent with the involvement of TRDMT1 in HR, loss of TRDMT1 increases the sensitivity of cells to radiation and PARP inhibitors (PARPi). Breast tumors expressing low levels of TRDMT1 are more responsive to radiotherapy, supporting the role of TRDMT1 in DSB repair. Importantly, depletion of TRDMT1 from tumors in mice increases their sensitivity to PARPi in vivo, showing that the function of m^5^C in HR is relevant to the use of PARPi in targeted cancer therapy. Together, our findings highlight the importance of a post-transcriptional DNA damage code on RNA, revealing a new layer of DNA repair regulation.

## Results

### DSBs induce m^5^C formation in mRNA at sites of DNA damage

**Fig. 1:**
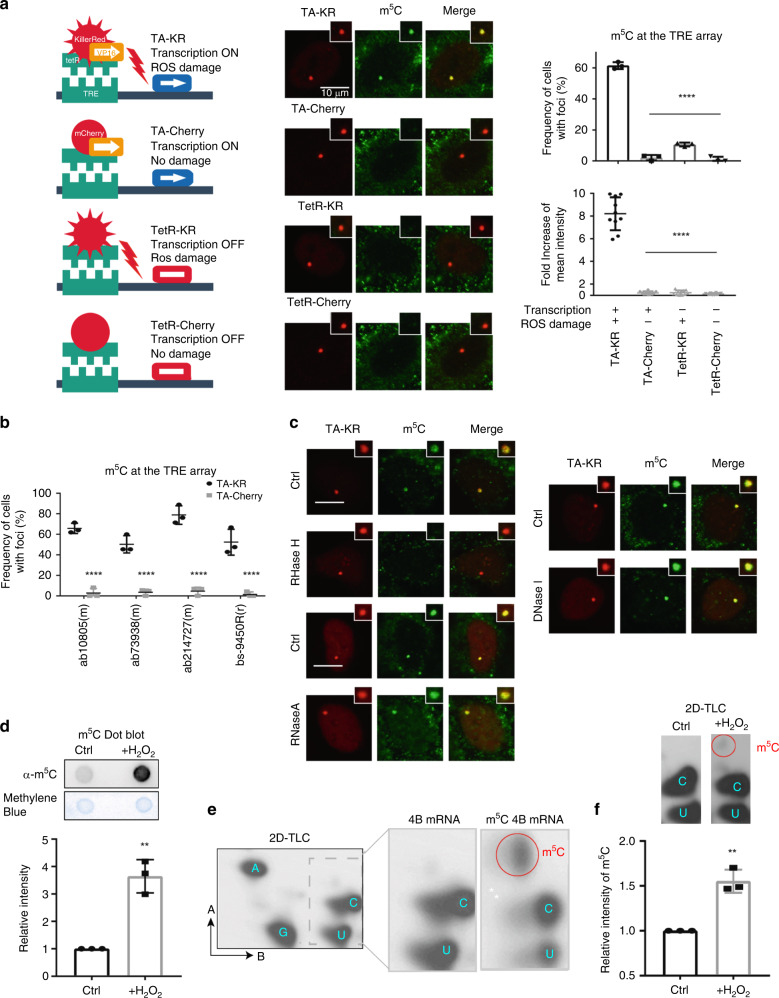
m^5^C mRNA methylation is enriched at transcriptionally active sites with DNA damage. **a** U2OS-TRE cells transfected with TA-KR/TA-Cherry/tetR-KR/tetR-Cherry plasmids were exposed to light for 30 min for KR activation and allowed to recover for 1 h before harvest (scale bar: 10 μm). Quantification of frequency of cells in 500 cells with m^5^C foci from three independent experiments, mean ± SD (upper right). Fold increase of m^5^C mean intensity = mean intensity of m^5^C at TA-KR/mean intensity of background (*n* = 20, mean ± SD) (lower right). **b** U2OS-TRE cells were transfected with TA-KR/TA-Cherry to induce local oxidative damage or for the control condition. Cells were then stained for m^5^C with four different anti-m^5^C antibodies. Frequency of m^5^C-positive cells in 500 cells was quantified (*n* = 3, mean ± SD). **c** U2OS-TRE cells transfected with TA-KR were digested with RNaseH1, RNaseA, or DNase I and stained for m^5^C quantification (scale bar: 10 μm). **d** The mRNA from Flp-in 293 cells treated with or without 2 mM H_2_O_2_ for 40 min was used for m^5^C measurement via dot blot. Quantification of m^5^C levels (mean ± SD) from three independent experiments normalized with Ctrl and methylene blue is shown. **e** ^32^P-labeled mRNA monophosphate nucleosides were run on 2D gels for 2D-TLC analysis. In vitro-transcribed 4B mRNA with or without m^5^C was run in parallel. Representative images from three sets of independent experiments are shown with arrows showing the directions of each solvent run. Position of each nucleotide and m^5^C are labeled (Left). **f** ^32^P-labeled mRNA monophosphate nucleosides from U2OS cells with or without 2 mM H_2_O_2_ for 40 min were run on 2D gels for 2D-TLC analysis. Representative images from three sets of independent experiments. Associated quantification of relative increase in m^5^C in peroxide-treated cells compared to control, normalized to nucleotide C (right). Statistical analysis was performed with the unpaired two tailed Student’s *t*-test. *p < 0.05; **p < 0.01; ***p < 0.001; ****p < 0.0001.

To investigate whether RNA is modified after DNA damage, we used a previously established approach to induce ROS locally at a specific genomic locus^8, 9^. A cassette containing an array of tetracycline responsive elements (TREs) and a downstream gene was stably integrated in the genome of U2OS cells (Fig. 1a). When a fusion protein of KR, tetR, and the transcription activator VP16 (TA-KR) is expressed in these cells, the protein binds to the TRE array and activates transcription locally. Upon light activation, TA-KR releases ROS and induces DNA damage at the locus, allowing us to analyze local DNA damage responses^8, 9^.

Using antibodies that specifically recognize m^5^C, we detected robust m^5^C signals at the TRE array in >60% of cells upon activation of TA-KR with light (Fig. 1a). Nuclear foci of γH2AX foci, a marker of DSBs, colocalized with m^5^C at the TRE array marked by TA-KR (Supplementary Fig. 1a), suggesting the induction of DSBs at this site. In contrast to TA-KR, fusion proteins that are unable to release ROS and/or activate transcription failed to induce m^5^C (Fig. 1a), suggesting that both DNA damage and transcription are required for the induction of m^5^C. The induction of m^5^C by TA-KR was observed using four independent anti-m^5^C antibodies (Fig. 1b, Supplementary Fig. 1b), ruling out the possibility of antibody-specific artifacts. Notably, the m^5^C signals at the TA-KR site were resistant to DNase I and RNaseA treatments but sensitive to RNaseH1 (Fig. 1c), suggesting the presence of m^5^C in the RNA in DNA:RNA hybrids. Consistent with the requirement of transcription for m^5^C formation, inhibition of RNA polymerase II by DRB or α-amanitin reduced the induction of m^5^C at the TA-KR site (Supplementary Fig. 1c). These results suggest that DNA damage in the TRE array induces m^5^C modification of the local RNA transcripts in DNA:RNA hybrids.

To test whether m^5^C is generally induced in mRNA after DNA damage, we isolated mRNA from cells untreated or treated with H_2_O_2_ or ionizing radiation (IR) and analyzed it with m^5^C antibody in dot blots (Fig. 1d, Supplementary Fig. 1d). An increase of m^5^C in mRNA was detected (Fig. 1d). The H_2_O_2_ induced m^5^C was reduced by RNaseH1 overexpression and DRB treatment before damage induction, confirming that m^5^C is formed in DNA:RNA hybrids in a transcription-dependent manner (Supplementary Fig. 1e). To confirm the induction of m^5^C in mRNA using an antibody-independent method, we used two dimensional thin-layer chromatography (2D-TLC) to analyze RNA modifications^19^. To determine the position of m^5^C in 2D-TLC, we compared the in vitro-transcribed 4B mRNA with and without m^5^Cs (Fig. 1e). One spot near C was specifically detected in the 4B mRNA modified by m^5^Cs. Importantly, the spot near C was also detected in the mRNA from H_2_O_2_-treated cells (Fig. 1f), supporting the idea that m^5^C is induced in mRNA by DNA damage.

Next, we characterized the kinetics of m^5^C accumulation at DNA damage sites (Supplementary Fig. 2a). We exposed cells to light for 30 min to activate TA-KR before we started the time course. At the beginning of the time course (0 h), ~30% of cells already displayed m^5^C foci because of the gradual activation of TA-KR. At 1 h into the time course, around 70% cells showed m^5^C foci. After the 1 h timepoint, m^5^C levels gradually declined. These data suggest that m^5^C formation peaks at DNA damage sites at ~1 h post damage induction. In contrast to m^5^C, m^6^A, another RNA modification that is rapidly induced by UV damage^20^, was not detected at the TA-KR site (Supplementary Fig. 2b) or in mRNA extracts after IR (Supplementary Fig. 1d), suggesting that m^6^A was either not induced by ROS damage or removed before the time course started. Importantly, the accumulation of m^5^C precisely correlated with the levels of R-loops at the TA-KR site over the time course (Supplementary Fig. 2a), supporting the notion that m^5^C is formed in DNA:RNA hybrids. Furthermore, the induction of m^5^C by TA-KR occurred similarly in cells arrested in G1, S, and G2/M phases of the cell cycle (Supplementary Fig. 2c), suggesting that this is a cell cycle-independent event.

Because ROS induce not only DSBs but also DNA single-strand breaks (SSBs) and oxidized bases, we asked whether DSBs are sufficient to induce m^5^C. Ninety-six copies of I-SceI sites were inserted to the TRE array and integrated in the genomes of U2OS cells^21^. Co-expression of the I-SceI endonuclease with TA-Cherry, which activates the transcription at the TRE array but does not induce DNA damage, induced m^5^C at the TRE array (Supplementary Fig. 2d), showing that I-SceI-generated DSBs are sufficient to trigger m^5^C formation. Consistent with the notion that DSBs promote m5C formation, the dose of H_2_O_2_ used to induce m^5^C (Fig. 1d) also triggered robust γH2AX formation (Supplementary Fig. 2e).

### TRDMT1 is a writer of RNA m^5^C at sites of DNA damage

**Fig. 2:**
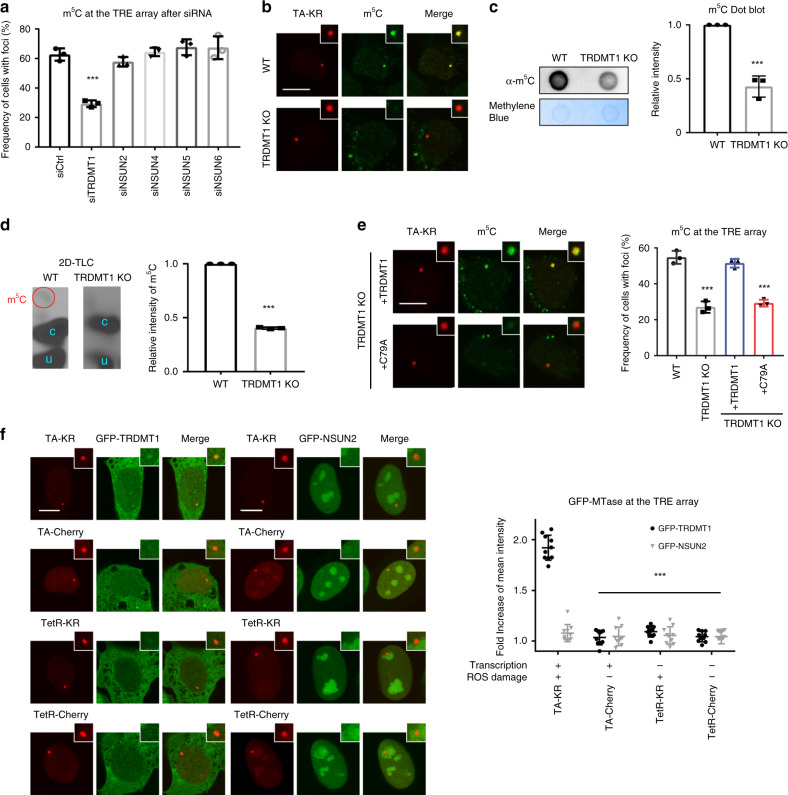
TRDMT1 mediates m^5^C mRNA methylation at DNA damage sites. **a** U2OS-TRE cells pre-treated with the indicated siRNA were transfected with TA-KR to induce local oxidative damage and stained for m^5^C. The frequency of cells in 500 cells with m^5^C foci (*n* = 3, mean ± SD) is shown. **b** The U2OS-TRE WT and TRDMT1 KO cells were transfected with TA-KR, stained for m^5^C, and quantified (scale bar: 10 μm) (*n* = 3, mean ± SD). **c** WT U2OS and TRDMT1 KO cells were treated with 2 mM H_2_O_2_ for 40 min. The mRNA was then extracted from the cell lysates for m^5^C measurement via dot blot. Quantification of m^5^C levels (mean ± SD) from three independent experiments normalized with Ctrl and methylene blue is shown. **d** ^32^P-labeled monophosphate nucleosides from mRNA from WT U2OS and TRDMT1 KO cells with or without 2 mM H_2_O_2_ for 40 min were run on 2D gels for 2D-TLC analysis. Representative images from three sets of independent experiments. Associated quantification of relative increases in m^5^C from H_2_O_2_-treated cells were compared to the control and normalized to nucleotide C (*n* = 3, mean ± SD). **e** The TRDMT1 KO and stably rescued cell lines expressing WT TRDMT1 or the C79A mutant were transfected with TA-KR, stained for m^5^C, and quantified (scale bar: 10 μm) (*n* = 3, mean ± SD). **f** U2OS-TRE cells transfected with TA-KR/TA-Cherry/tetR-KR/tetR-Cherry and GFP-TRDMT1/GFP-NSUN2 plasmids were light-irradiated and allowed to recover for 1 h before fixation and were quantified (scale bar: 10 μm) (*n* = 10, mean ± SD). Statistical analysis was performed with the unpaired two tailed Student’s *t*-test. *p < 0.05; **p < 0.01; ***p < 0.001; ****p < 0.0001.

Having found that m^5^C is a local marker of DSBs, we asked which enzyme is responsible for this RNA modification after DNA damage. We used siRNAs to individually knockdown all RNA methyltransferases known to generate m^5^C (Fig. 2a, Supplementary Fig. 3a)^22^. Depletion of only one RNA methyltransferase in this group, TRDMT1, significantly reduced TA-KR-induced m^5^C. To confirm the result of TRDMT1 knockdown, we generated independent TRDMT1 knockout (KO) cell lines using CRISPR/Cas9 (Supplementary Fig. 3b). Consistent with a role of TRDMT1 in damage-induced m^5^C formation, TA-KR-induced m^5^C was reduced in TRDMT1 KO cells (Fig. 2b). We also isolated mRNA from H_2_O_2_-treated wild-type (WT) and TRDMT1 KO cells and analyzed it with m^5^C dot blot and 2D-TLC. Both dot blot and 2D-TLC confirmed that m^5^C was reduced in the mRNA of TRDMT1 KO cells (Fig. 2c, d). Importantly, expression of WT TRDMT1 (TRDMT^WT^) but not the catalytic mutant (TRDMT1^C79A^)^16^ in KO cells rescued TA-KR-induced m^5^C (Fig. 2e), showing that TRDMT1 activity is important for damage-induced m^5^C formation.

To understand how TRDMT1 is regulated by DNA damage, we used GFP-tagged TRDMT1 to study its localization. GFP-TRDMT1 was recruited to the TRE array in a DNA damage- and transcription-dependent manner (Fig. 2f), which parallels with the formation of m^5^C at this locus (Fig. 1a). In contrast to TRDMT1, NSUN2, another methyltransferase known to generate m^5^C in RNA^22^, did not localize to this locus after DNA damage (Fig. 2f). Similar to the formation of m^5^C, the recruitment of GFP-TRDMT1 to the TA-KR site is cell cycle-independent (Supplementary Fig. 3c). Furthermore, when m^5^C was induced by the I-SceI-generated DSBs in the TRE array, GFP-TRDMT1 was recruited to this locus (Supplementary Fig. 3d). These results suggest that TRDMT1 is present at DNA damage sites when m^5^C is formed.

To test whether TRDMT1 binds RNA upon DNA damage, we used 5-azacytidine (5-Aza) to trap cytidine methyltransferases on DNA and RNA^23^. After 5-Aza treatment, the amount of RNA captured by GFP-TRDMT1 was increased in H_2_O_2_-treated cells (Supplementary Fig. 3e), suggesting that the association of TRDMT1 with RNA is increased after DNA damage. Similar to the formation of m^5^C at the TA-KR site, the accumulation of GFP-TRDMT1 at this locus was reduced by WT RNaseH1 but not the inactive mutant (Supplementary Fig. 4a). Furthermore, inhibition of RNA polymerase II also reduced the recruitment of GFP-TRDMT1 (Supplementary Fig. 4b). These results suggest that TRDMT1 is recruited in a transcription- and R-loop-dependent manner. Consistent with this possibility, we found that purified TRDMT1 bound to DNA:RNA hybrids directly in vitro (Supplementary Fig. 4c), suggesting that TRDMT1 is recruited to DNA damage sites by R-loops.

### TRDMT1 is required for efficient HR

**Fig. 3:**
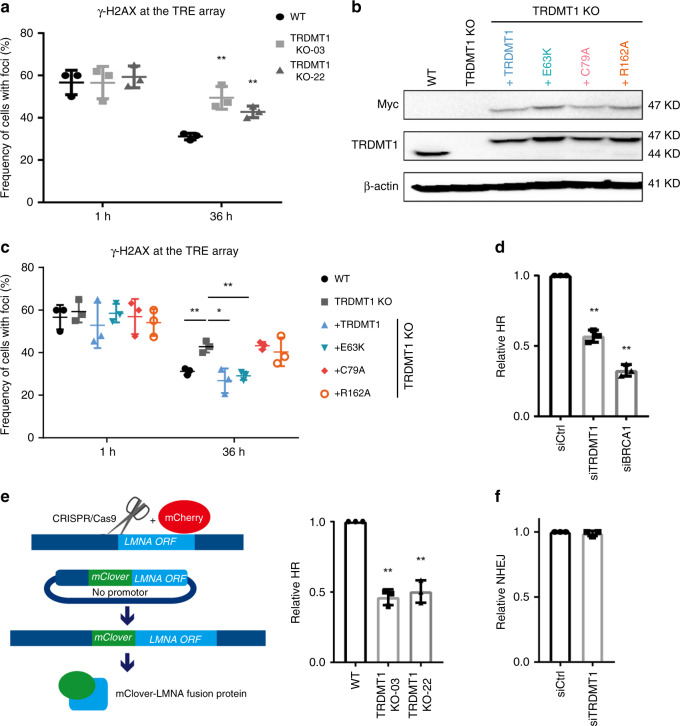
TRDMT1 is required for damage removal and HR. **a** WT or TRDMT1 KO U2OS-TRE cells were transfected with TA-KR and stained for γH2AX at the indicated timepoint after damage (*n* = 3, mean ± SD). **b** Stable expression of Myc-tagged TRDMT1 in TRDMT1 KO U2OS-TRE cell lines shown in Western blots. Expression of β-actin is shown as a positive control. **c** U2OS-TRE WT, TRDMT1 KO, and TRDMT1 stably expressing cells were stained for γH2AX after damage caused by TA-KR at the indicated timepoint (*n* = 3, mean ± SD). **d** DR-GFP cells were pre-treated with siTRDMT1, siBRCA1, or control siRNA and then transfected with the NLS-I-SceI plasmid to induce DSBs. The GFP-positive population was analyzed by flow cytometry (*n* = 3, mean ± SD). **e** WT or TRDMT1 KO U2OS cells were co-transfected with CRISPR/Cas9-sgLMNA, LMNA-mClover, and mCherry plasmids. The fraction of mClover-positive cells in the mCherry-positive population was analyzed by flow cytometry (*n* = 3, mean ± SD). **f** EJ5-GFP cells were pre-treated with siTRDMT1 or control siRNA and then transfected with the NLS-I-SceI plasmid to induce DSBs. The GFP-positive population was analyzed by flow cytometry (*n* = 3, mean ± SD). Statistical analysis was performed with the unpaired two tailed Student’s *t*-test. *p < 0.05; **p < 0.01; ***p < 0.001; ****p < 0.0001.

To test whether TRDMT1 is important for DSB repair, we compared the clearance of TA-KR-induced γH2AX at the TRE array in WT and TRDMT1 KO cells. γH2AX was similarly induced by TA-KR in WT and KO cells at 1 h after light activation (Fig. 3a). However, at 36 h after damage induction, the levels of γH2AX were significantly higher in TRDMT1 KO cells than in WT cells, suggesting that loss of TRDMT1 impairs DSB repair. Importantly, expression of TRDMT1^WT^ in KO cells significantly rescued DSB repair (Fig. 3b, c). In contrast to TRDMT1^WT^, a TRDMT1 mutant defective in both catalytic activity and RNA binding (TRDMT1^C79A^) and a mutant defective only for catalytic activity (TRDMT1^R162A^)^16^ failed to rescue DSB repair (Fig. 3b, c). A third TRDMT1 mutant (TRDMT1^E63K^) with a higher affinity for RNA than TRDMT1^WT^ restored repair efficiently (Fig. 3b, c)^17^. These results suggest that the activity of TRDMT1 is required for the efficient repair of ROS-induced DSBs.

We recently showed that ROS-induced DSBs are repaired through a BRCA1/2-independent but RAD51/RAD52-dependent non-canonical HR pathway^9^, possibly because the functions of BRCA1/2 are inhibited by ROS-induced lesions or repair intermediates in this context. Next, we asked whether TRDMT1 has a general function in different DSB repair pathways. Using the direct repeat *GFP* (DR-GFP) assay, we found that knockdown of TRDMT1 reduced the repair of I-SceI-generated DSBs (Fig. 3d). Furthermore, the HR-mediated integration of *mClover* to a site of CRISPR/Cas9-generated DSBs in the *LAMIN A* gene was reduced in TRDMT1 KO cells compared to WT cells (Fig. 3e). These results suggest that TRDMT1 is also involved in the BRCA1/2-dependenet canonical HR pathway. TRDMT1 knockdown out did not alter the cell cycle (Supplementary Fig. 5a), ruling out indirect effects of cell-cycle alterations. In contrast to its effects in HR reporter assays, TRDMT1 loss did not affect the efficiency of non-homologous end joining (NHEJ) in the reporter assay (Fig. 3f). These results suggest that TRDMT1 promotes DSB repair through both canonical and non-canonical HR pathways.

Consistent with the role of TRDMT1 in HR, TRDMT1 KO cells were more sensitive to IR than U2OS WT cells (Supplementary Fig. 5b). DRB treatment also sensitizes U2OS cells to IR but don’t further sensitize TRDMT1 KO cells (Supplementary Fig. 5c). The IR sensitivity was suppressed by TRDMT1^WT^ and TRDMT1^E63K^, but not TRDMT1^C79A^ and TRDMT1^R162A^ (Supplementary Fig. 5b). Interestingly, TRDMT1^R162A^ localized to the TRE array efficiently (Supplementary Fig. 5d) but failed to suppress the IR sensitivity of KO cells, suggesting that the catalytic activity of TRDMT1 is required at DSBs to promote HR.

TRDMT1 is known to modify tRNA in the cytoplasm, but a fraction of TRDMT1 is detected in the nucleus. Our finding that TRDMT1 functions at DSBs to promote HR raises a question of whether the nuclear and cytoplasmic functions of TRDMT1^18^ can be separated. To address this, we tagged TRDMT1 with either a nuclear export signal (NES) or a nuclear localization signal (NLS). Although NES-TRDMT1 was an exclusively cytoplasmic protein, NLS-TRDMT1 was readily detected in the nucleus (Supplementary Fig. 5e). In TRDMT1 KO cells, only NLS-TRDMT1 was localized to TA-KR sites (Supplementary Fig. 5e). In contrast to NLS-TRDMT1, NES-TRDMT1 did not restore m^5^C formation and γH2AX clearance at the locus marked by TA-KR (Supplementary Fig. 5f, g), nor did it suppress IR sensitivity (Supplementary Fig. 5h). These results suggest that the nuclear function of TRDMT1 in DNA repair is distinct from its cytoplasmic function in tRNA regulation. Moreover, TRDMT1 knockdown delayed the clearance of γH2AX foci after IR and reduced RAD51 foci without affecting RAD51 and RAD52 levels (Supplementary Fig. 6a–d), supporting the notion that the role of TRDMT1 in DNA repair is independent of it function in protein translation.

### RAD52 is a reader of RNA m^5^C

**Fig. 4:**
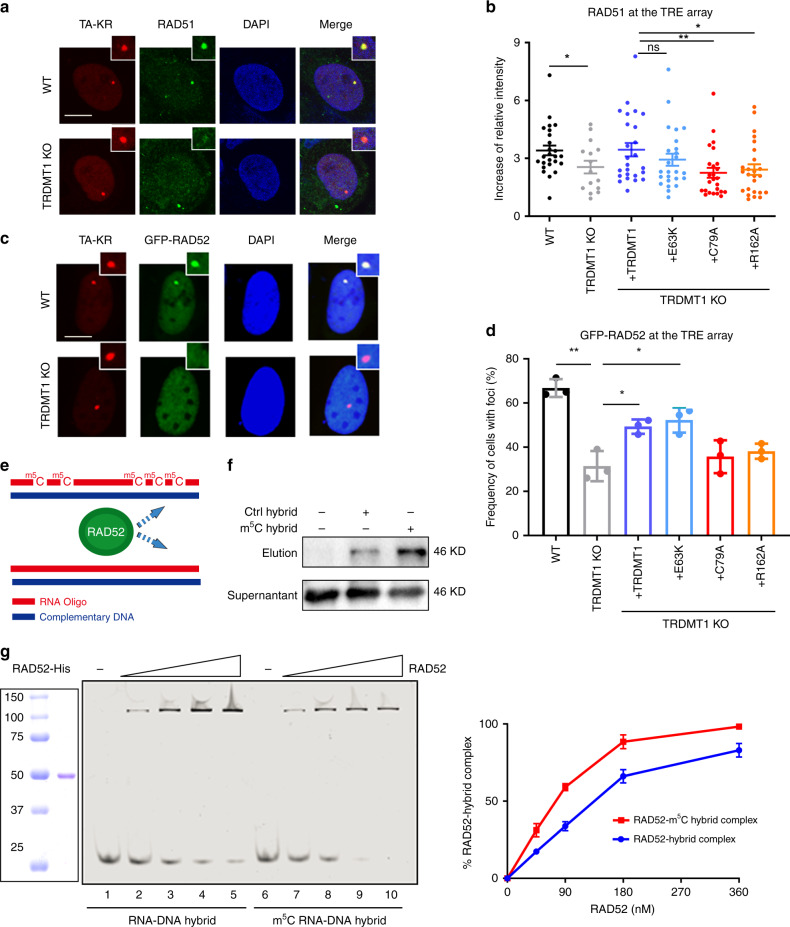
RAD52 is a m^5^C reader. **a** U2OS-TRE WT and TRDMT1 KO cells were transfected with TA-KR and stained for RAD51 1 h after light irradiation. Representative figures were shown (scale bar: 10 μm). **b** U2OS-TRE WT, TRDMT1 KO, and TRDMT1 stably rescued cell lines were transfected with TA-KR. and stained for RAD51 1 h after light irradiation. Fold increase of RAD51 foci intensity was calculated (*n* = 25, mean ± SD). **c** U2OS-TRE WT and TRDMT1 KO cells were transfected with TA-KR and GFP-RAD52 and fixed 1 h after light irradiation. Representative figures were shown (scale bar: 10 μm). **d** U2OS-TRE WT, TRDMT1 KO, and TRDMT1 stably rescued cell lines were co-transfected with GFP-RAD52 and TA-KR and quantified for RAD52 foci frequency (*n* = 3, mean ± SD). **e** RNA oligos with or without m^5^C modification and their complementary DNA oligos were chemically synthesized for RAD52 binding experiments. **f** RAD52 protein in the cell lysate was pulled down by biotin-labeled DNA:RNA hybrids, with or without m^5^C RNA modification, coated on streptavidin magnetic beads. **g** The binding of RAD52 protein to DNA:RNA hybrids with or without m^5^C RNA modification was measured in electrophoretic mobility shift assays and quantified (*n* = 3, mean ± SD). Statistical analysis was performed with the unpaired two tailed Student’s *t-*test. *p < 0.05; **p < 0.01; ***p < 0.001; ****p < 0.0001.

The requirement of TRDMT1 for the repair of ROS-induced DSBs prompted us to test whether the repair proteins involved in this process are readers of m^5^C. Consistent with the role of TRDMT1 in the repair of ROS-induced DSBs, the damage-induced localization of RAD51 to the TRE array was reduced in TRDMT1 KO cells (Fig. 4a). The catalytic activity of TRDMT1 is required for the localization of RAD51 (Fig. 4b). RAD52, which is required for the recruitment of RAD51 to the TRE array, also depends on the activity of TRDMT1 to localize to this ROS-damaged locus (Fig. 4c, d). Thus, the RAD52–RAD51 axis involved in the repair of ROS-induced DSBs is regulated by m^5^C.

RAD52 is known to bind DNA:RNA hybrids^9, 24, 25^. To test whether m^5^C increases the affinity of RAD52 to DNA:RNA hybrids, we synthesized a set of biotin-labeled RNA oligos of 30 or 50 nucleotides with or without five m^5^Cs to generate DNA:RNA hybrids (Fig. 4e). The 30-bp DNA:RNA hybrid containing five m^5^Cs captured RAD52 protein in cell lysates more efficiently than the hybrid containing unmodified RNA (Fig. 4f). Furthermore, in an electrophoresis mobility shift assay, the 50-bp DNA:RNA hybrid containing five m^5^Cs bound to purified RAD52 with a higher efficiency than the unmodified hybrid (Fig. 4g). These results suggest that RAD52 is a reader of the m^5^C in DNA:RNA hybrids, providing an explanation for how TRDMT1 and m^5^C promote the recruitment of RAD52 to ROS-induced DSBs. Furthermore, ALYREF, a known reader of tRNA m^5^C^26^, was not recruited to the TA-KR site as RAD52 (Supplementary Fig. 6e), suggesting that RAD52 is specific reader of the m^5^C at DNA damage sites.

### TRDMT1 contributes to the resistance of cancer cells to radiotherapy and PARP inhibitors

**Fig. 5:**
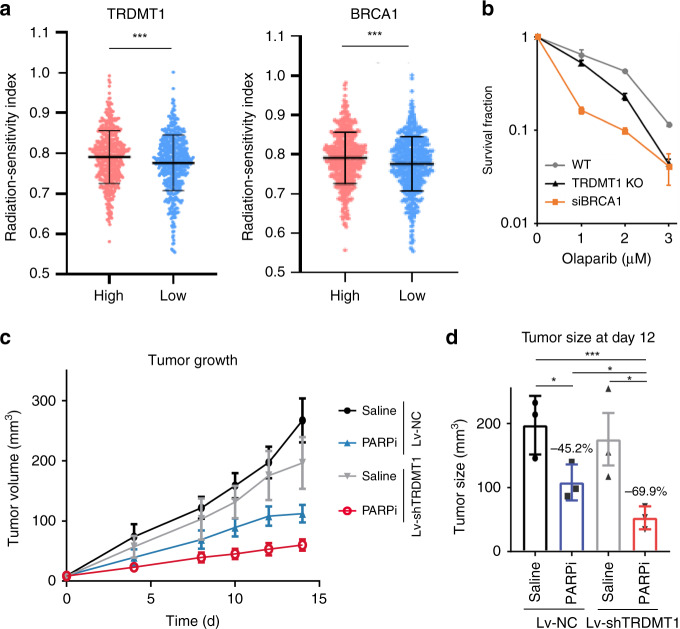
TRDMT1 inhibition sensitizes cells to radiation and PARPi. **a** Clinical and RNA-seq gene expression data were downloaded from TCGA. RSI was calculated by a rank-based linear regression algorithm. The median value of BRCA1 and TRDMT1 TPM was defined as the cutoff to classify patients into high expression and low expression groups (*n* = 1091, mean ±SD). **b** WT, TRDMT1 KO U2OS, or siBRCA1 cells were treated with Olaparib at the indicated dose. The survival rate was measured via the colony formation assay (*n* = 3, mean ± SEM). **c** Tumor volume in mice injected with LV-shTRDMT1 or LV-NC pre-treated MDA-MB-231 cells at the indicated time (*n* = 3, mean ± SEM). One week after injection, 50 mg/kg Olaparib or saline was intraperitoneally injected into the xenograft tumors once every other day. **d** Tumor volume in mice injected with LV-shTRDMT1 or LV-NC pre-treated MDA-MB-231 cells at day 12 after PARPi treatment (*n* = 3, mean ± SEM). Statistical analysis was performed with the unpaired two tailed Student’s *t*-test. *p < 0.05; **p < 0.01; ***p < 0.001; ****p < 0.0001.

The role of TRDMT1 in HR prompted us to investigate whether it is relevant to the radiation response of tumors in patients. We analyzed breast cancer patient radiosensitivity index (RSI) generated from the RNA-seq data and clinical information in the TCGA database^27, 28^. Low RSI correlates with high radiosensitivity in patients; for example, BRCA1-low patients showed lower RSI than BRCA1-high patients (Fig. 5a). We classified breast cancer patients from the TCGA database into TRDMT1-low and -high groups using the median value of TRDMT1 transcripts per million (TPM) as the cutoff value. The TRDMT1-low group showed a lower RSI than the TRDMT1-high group (Fig. 5a), supporting the idea that low expression of TRDMT1 in tumors is associated with a better response to radiotherapy.

HR-deficient cells are highly sensitive to PARP inhibitors (PARPi)^29–31^. Given that the function of TRDMT1 in HR, we tested whether loss of TRDMT1 renders cells sensitive to PARPi like BRCA1 suppression (Fig. 5a). Indeed, TRDMT1 KO cells were more sensitive to the PARPi Olaparib than WT cells (Fig. 5b). Moreover, knockdown of RAD52 also increased Olaparib sensitivity. Importantly, knockdown of RAD52 in TRDMT1 KO cells did not further increase Olaparib sensitivity, which suggests that TRDMT1 and RAD52 are epistatic (Supplementary Fig. 6f). MDA-MB-231 cells are HR-defective due to the high expression of Polθ, an inhibitor of HR^32^. MDA-MB-231 cells infected with lentiviruses (LV)-expressing TRDMT1 shRNA (shTRDMT1) or control shRNA (NC) were injected intraperitoneally into mice, and tumor growth was measured over 14 days. As expected, PARPi significantly reduced the growth of tumors treated with control shRNA (LV-NC) (Fig. 5c, d, Supplementary Fig. 7a–c). In the absence of PARPi, depletion of TRDMT1 (LV-shTRDMT1) also reduced tumor growth modestly (Fig. 5d, Supplementary Fig. 7a, b). Notably, on day 12 after the injection of tumor cells, PARPi reduced the growth of TRDMT1-depleted tumors more than control tumors (70% vs. 45%) (Fig. 5d), suggesting that loss of TRDMT1 increases the HR deficiency in tumor cells.

## Discussion

The proper repair of DSBs requires efficient recruitment of DNA repair proteins to sites of DNA damage. The chromatin flanking DSBs has been shown to have a critical role in this process. For example, the γH2AX in the chromatin flanking DSBs promotes the recruitment of MDC1, 53BP1, and BRCA1 through a cascade of phosphorylation- and ubiquitination-mediated protein interactions^33, 34^. During this process, the protein PTMs that are locally induced at sites of damage serve as DNA damage codes to recruit specific repair proteins. A number of protein kinases and ubiquitin ligases function as writers of DNA damage codes, whereas a group of DNA repair proteins act as readers through their phospho- or ubiquitin-binding domains^35, 36^. Recent studies by others and us suggested that RNAs also have an important role in the recruitment of specific repair proteins^37, 38^. Some of the repair proteins recruited by RNAs, such as CSB and RAD52, have the ability to bind DNA:RNA hybrids, which are induced by DSBs^9^. However, it is still not known whether the functions of RNAs in DSB repair are also regulated by DNA damage codes.

In this study, we find that the RNA modification m^5^C is locally induced by DSBs at sites of DNA damage. Interestingly, a recent study showed that another RNA modification, m^6^A, was induced by UV damage but not DSBs^39^. Although we did not detect m6A at the TA-KR site, we do not exclude the possibility that m^6^A is transiently induced before significant m^5^C accumulation. Together with this study, our results suggest that m^5^C is a specific DNA damage code of DSBs. We find that m^5^C is formed in mRNA in response to DSBs. Furthermore, m^5^C is present in DNA:RNA hybrids at DNA damage sites. It is plausible that DSBs trigger the hybridization of mRNA with DNA template locally, and the RNA in DNA:RNA hybrids is subsequently modified by m^5^C to promote the recruitment of repair proteins. This mechanism may ensure that m^5^C is specifically generated in the RNA at sites of DSBs.

Our data suggest that TRDMT1 is the RNA methyltransferase responsible for damage-induced m^5^C formation. TRDMT1 is best known for its role in tRNA regulation in the cytoplasm^18^. We find that the nuclear location of TRDMT1 is critical for its function in DSB repair, suggesting that this function of TRDMT1 is distinct from its cytoplasmic role in tRNA regulation. In cells, TRDMT1 localizes to sites DNA damage in a manner dependent on DNA:RNA hybrids. In vitro, TRDMT1 has the ability to bind DNA:RNA hybrids directly. These findings suggest that TRDMT1 is recruited to the DNA:RNA hybrids at sites of DNA damage, where it modifies the RNA in DNA:RNA hybrids and generates m^5^Cs. It is important to demonstrate the methyltransferase activity of TRDMT1 on DNA:RNA hybrids in future studies, which will firmly establish TRDMT1 as the writer of m^5^C at DSBs. Erasers of RNA modifications, such as the obesity-associated protein FTO^40^, may also be important for the regulation of DNA repair. Future studies are needed to investigate this possibility.

TRDMT1 is important for HR not only at nuclease-generated DSBs but also at ROS-induced DSBs, suggesting that TRDMT1 and m^5^C are generally required for efficient HR. Consistent with this idea, cells lacking TRDMT1 are sensitive to IR. In the context of ROS-induced DSBs, TRDMT1 is required for the localization of RAD51 and RAD52 to sites of DNA damage. We previously showed that RAD52 binds DNA:RNA hybrids with help of CSB and promotes RAD51 localization to DNA damage sites^9^. Our data in this study show that RAD52 preferentially binds to DNA:RNA hybrids containing m^5^C-modified RNA, suggesting that RAD52 is a reader of m^5^C at DNA damage sites. Thus, the results of this study reveal a previously unknown TRDMT1–m^5^C–RAD52–RAD51 axis that promotes the non-canonical HR repair of ROS-induced DSBs. This axis provides an example of how a DNA damage code is written on RNA at DNA damage sites and how this code is read by DNA repair proteins, showing that the post-transcriptional modifications of RNA can also serve as DNA damage codes to regulate DNA repair. It should be noted that the RAD52 is unlikely the only reader of m^5^C in the HR pathway. Although RAD52 is recruited in a TRDMT1-dependent manner in cells and is a reader of m^5^C in vitro, we do not exclude the possibility that RAD52 has affinity to other RNA modifications. TRDMT1 is required for the efficient repair of nuclease-generated DSBs, which primarily relies on BRCA1/2 but not RAD52, suggesting that additional readers of m^5^C likely exist in the canonical HR pathway.

The roles of TRDMT1 and m^5^C in HR are relevant to the sensitivity of cancer cells to radiotherapy and PARPi therapy. Similar to BRCA1 deficiency, low expression of TRDMT1 in breast tumors may induce genomic instability but also render tumors responsive to radiotherapy. In addition, low TRDMT1 expression in tumors may compromise HR and provide an opportunity for PARPi therapy. Our data show that HR-defective tumors are particularly sensitive to concomitant TRDMT1 loss and PARP inhibition, raising the possibility that inhibitors of TRDMT1 may sensitize HR-deficient tumors to PARPi and/or overcomes PARPi resistance. The TRDMT1–m^5^C axis discovered in this study extends the concept of DNA damage codes from post-translational modifications of proteins to post-transcriptional modifications of RNAs, providing exciting opportunities for targeted cancer therapy.

## Methods

### Cell culture and transfection

U2OS, Flp-in 293, and 293FT cells were cultured in Dulbecco’s modified Eagle medium (DMEM, Cat#12-604F, Lonza; Basel, Switzerland) with 10% (vol/vol) FBS at 37 °C with 5% CO_2_. The U2OS-TRE cells used for the DART system have been described in previous articles^8, 9^. For plasmid and siRNA transfection, Lipofectamine 2000 and Lipofectamine RNAiMax (Invitrogen; Carlsbad, CA, USA) were used following a manufacture’s standard protocol. The siRNA for TRDMT1 was purchased from Invitrogen (siRNA ID: s4219, Cat#: 4392420). Other siRNAs include NSUN2 (M-018217-01), NSUN4 (D-027291-01-0002), NSUN5 (D-021349-17-0002), and NSUN6 (D-018822-01-0002).

### Microscopy and activation of KR

The Olympus FV1000 confocal microscopy system (Cat#: F10PRDMYR-1, Olympus; Waltham, MA, USA) and FV1000 software were used for acquisition of images. Cells were cultured in 35-mm glass-bottom dishes (P35GC-1.5-14-C, MatTek; Ashland, MA, USA) before observation. Activation of KR in bulky cells was completed by exposing them to a 15-W Sylvania (Wilmington, MA, USA) cool white fluorescent bulb for 25 min in a UVP stage. The intensity was measured by ImageJ 1.50i software. *p*-values were calculated by the Student’s *t*-test.

### Immunoassays and m^5^C staining

Cells for immunofluorescence (IF) observation were fixed in 4% PFA (19943 1 LT, Affymetrix/ThermoFisher Scientific) for 15 min at room temperature and further treated with 0.2% Triton X-100 for 10 min. They were then blocked by 3% BSA (A-7030, Sigma-Aldrich) for 30 min at room temperature or not blocked. Primary antibodies were diluted in DMEM without FBS and incubated with cells overnight at 4 °C. The samples were then washed three times with 0.05% PBST, and the cells were incubated with secondary antibodies for 1 h at room temperature followed by three washes with 0.05% PBST. Incubation with (1:1000 dilution) DAPI for 10 min at room temperature was optional.

For m^5^C staining using the heat method, cells were fixed and permeabilized in a 35-mm glass-bottom dish, incubated in buffer (10 mM Tris-HCl, 2 mM EDTA, pH 9), and steamed on a 95 °C heating block for 20 min to expose the antigen. The dish was cooled, washed three times by PBS and blocked using 5% BSA in 0.1% PBST for 0.5 h at room temperature. The primary and secondary antibodies were diluted in the same buffer (5% BSA in 0.1% PBST) and followed the standard IF protocol, which is modified from the classical heat-induced antigen retrieval method for PFA-fixed tissues using Tris-EDTA buffer. The S9.6 staining was done using the same heat method.

For m^5^C staining with RNaseH1 overexpression, U2OS-TRE cells were co-transfected with TA-KR and HA-RNaseH1. The m^5^C antibody was co-stained with HA antibody (ab9110). The m^5^C foci on TA-KR in cells highly expressing HA-RNaseH1 were examined.

Exogenous nuclease treatments for m^5^C staining were done after fixation and permeabilization. For RNaseA treatment: after heat treatment, cells were incubated with 100 μg/mL RNaseA in 100 μL RNase digestion buffer (5 mM EDTA, 300 mM NaCl, 10 mM Tris-HCl, pH 7.5) at room temperature for 25 min. For RNaseH1 treatment, the cells were incubated with 15 U RNaseH1 (Cat#: EN0201, ThermoFisher Scientific) in 100 μL reaction buffer (200 mM Tris-HCl, pH 7.8, 400 mM KCl, 80 mM MgCl_2_, 10 mM DTT) at room temperature for 25 min. For DNase I treatment, cells were incubated with 20 U (1 μL) DNase I in 100 μL buffer (10 mM Tris-HCl, 2.5 mM MgCl_2_, 0.5 mM CaCl_2_, pH 7.5) at 37 °C for 30 min followed by heat treatment. After the nuclease treatments, the cells were blocked with 5% BSA in 0.1% PBST buffer and stained with m^5^C antibody.

### Dot blot assay

Total poly(A) + mRNA from U2OS-TRE or Flp-in 293 cells was purified with a Dynabeads™ mRNA DIRECT™ Purification Kit (Cat#: 61011, ThermoFisher Scientific, Waltham, MA, USA). The same amount of mRNA from different samples was diluted to the same concentration by 10 mM Tris-HCl from the kit. The mRNA solutions were loaded on a positive-charged Nylon66 membrane (Biodyne B transfer membrane, 0.45 μm, 60209), and linked by a UV Stratalinker 2400 (Stratagene; La Jolla, CA, USA) at 1200 μJ twice. Then, the membrane was washed in 0.02% PBST for 10 min. Primary antibody was diluted 1:100 in 5% non-fat milk in 0.02% PBST. After overnight incubation at 4 °C, the membrane was washed twice gently in 0.02% PBST for 10 min. Secondary antibody was diluted 1:10,000 in 5% non-fat milk in 0.02% PBST and then washed three times. The membrane was stained by 0.1% methylene blue (Cat#: M9140-25G, Sigma-Aldrich; St. Louis, MO, USA) in 0.5 M NH_4_OAc. Antibodies used in this study are summarized in Supplementary Table 1.

### 2D-TLC assay

4B mRNA in RNA with a firefly luciferase reporter containing 3′UTR 4 microRNA sites to an artificial microRNA was used as a control. No microRNAs or regulators of 4B mRNA are known^41^. In vitro-transcribed 4B mRNA with or without m^5^C was run in parallel. Poly(A)+ mRNA from Flp-in 293 cells before and after induced DNA damage was purified with a Dynabeads™ mRNA DIRECT™ Purification Kit (Cat#: 61011, ThermoFisher Scientific). 2D-TLC was performed as described previously^19, 42^. Approximately 500 ng of RNA was digested with RNase If and RNase T2 (Cat#: M0243L, New England Biolabs/NEB; Ipswich, MA, USA and Cat#: LS01502, Worthington Biochemical Corp.; Lakewood, NJ, USA), followed by labeling with γ-P^32^-ATP and T4 polynucleotide kinase (Cat#: M0201L, NEB). The reactions were further processed and digested with P1 Nuclease (Cat#: M0660S, NEB). The reactions were loaded on cellulose TLC plates (20 × 20 cm, EMD Millipore™ Precoated TLC and PLC Glass Plates, Cat#: M1057160001, ThermoFisher Scientific) and developed in two solvent systems (Fig. 1b, arrows): solvent A with isobutyric acid: 0.5 M NH_4_OH (5:3 v/v; Cat#: AAL04038AP, AC423305000) for the first dimension and solvent B with phosphate buffer/ammonium sulfate/*n*-propanal (100/60/2 (v/w/v)) for the second dimension and then exposed to film. The spots were quantified with ImageJ software and normalized for total input cpms for comparison between samples.

### Cell synchronization and cell-cycle analysis

The U2OS-TRE cells were synchronized to different phases using double thymidine block method. After 1 day of cell plating, 2 mM thymidine was added to the medium for 16 h. Then thymidine was removed from the medium by washing the cells with PBS three times. After 8 h cultivation without thymidine, the cells were incubated with 2 mM thymidine again for 16 h. 0 h (G1phase), 4 h (S phase), and 8 h (G2/M phase) cultivation after thymidine removal, cells were collected for cell-cycle analysis

For cell-cycle analysis, cells were collected and washed with PBS and then fixed in 70% ethanol overnight at 4 °C. After wash once with 2% BSA, the cell pellet was suspended in 2% BSA with 50 μg/mL propidium iodide and 100 μg/mL RNaseA. After 30 min incubation at 37 °C in dark, the cells were applied to flow cytometry analysis.

### Plasmids

The TA-KR, tetR-KR, TA-Cherry, and tetR-Cherry on pBroad3 plasmids were constructed and described in a previous publication^43^. The GFP-NSUN2 and GFPspark-TRDMT1 constructs were purchased from OriGene (RG214459; Rockville, MD, USA) and Sino Biological (HG11224-ACG, Beijing, China). The TRDMT1 gene was then subcloned to the EGFP-C3 plasmid (Clontech/Takara Bio USA; Mountain View, CA, USA) linked by *Eco*RI and *Bam*HI for imaging experiments. For CRISPR-Cas9 knockout, the sgRNA sequence was integrated into the PX330 plasmid (provided by Feng Zhang). The Myc-tagged TRDMT1 mutations were subcloned via overlapping PCR to the pLVX-puro plasmid (Clontech) by *Eco*RI and *Not*I. The NLS-GFP-RAD52, Flag-RAD51, NLS-I-SceI^8^, and HA-RNaseH1-WT/D210N^44^ plasmids have been described previously. The Myc-NLS-TRDMT1 and Myc-NES-TRDMT1 in PLVX-IRES-Puro vectors were constructed according to previous literature^18^. An NLS from the SV40 T antigen, PKKKRKV, or an NES from MAPKK, LQKKLEELEL, was added to the N terminus of TRDMT1 by PCR. The primers used to generate the TRDMT1 mutants are listed in Supplementary Table 2.

### Western blots

For Western blot analysis, samples were boiled at 95 °C for 5–8 min in SDS loading buffer, subjected to electrophoresis in 10–12% SDS-polyacrylamide gels, and transferred to PVDF membranes. The membranes were previewed with Ponceau S Red solution (Cat#: P3504-10G, Sigma-Aldrich). For block and antibody dilution, 5% non-fat milk in PBS was used. After primary antibody incubation at 4 °C overnight and secondary antibody incubation at room temperature for 1 h, the membranes were washed in 0.1% PBST three times. Chemiluminescent HRP substrate was purchased from Millipore (Cat#: WBKLS0500; Burlington, MA, USA). Images were taken in the BIO-RAD (Hercules, CA, USA) Universal Hood II machine with corresponding ImageLab software.

### CRISPR-Cas9 KO generation

Oligonucleotides were designed for TRDMT1 KO in the human genome, whose map is shown in Supplementary Fig. 3b, that target the following sequences: upstream 5′-GACCGGCAGGCCTAGCTCCG-3′, and downstream 5′-GTTGGGAGTCGGGATTCGCA-3′ synthesized by Integrated DNA Technologies (IDT; Coralville, IA, USA). They were inserted into a PX330 plasmid and transfected into U2OS-TRE or Flp-in 293 cells. Primers designed for genotyping PCR were forward primer 5′-GCCTTATTGTTTTCCGTCCTTTGTTC-3′ and reverse primer 5′-AAGCCTGTTTGTCATTCTGTATCCCC-3′. 48 h after transfection, single cells were separated in 96-well dishes to obtain monoclonal colonies.

### Construction of TRDMT1 mutant stable expression cell lines

The U2OS-TRE TRDMT1-stable cells were constructed by LV infection. The cDNA of WT and mutated TRDMT1 were subcloned from the EGFP-C3 vector into the pLVX-IRES-Puro Vector with an N-terminal Myc tag. The plasmids were co-transfected into 293FT cells for virus packaging. Culture medium was changed 8 h after transfection. 48 h later and the medium was collected and filtered with a 0.45-μm filter (Millex-HA, SLHAM33SS; Sigma-Aldrich). The U2OS-TRE TRDMT1 KO cells were cultured in the medium mixed with normal DMEM (10% FBS) at a 1:1 ratio. Polybrene (10 μg/mL) was added to the culture system to promote efficiency. 48 h later, the cells were cultured in DMEM (with 10% FBS) with 1 μg/mL puromycin, and the medium was changed once every 2 days.

### Azacytidine-IP

TRDMT1 KO + GFP-WT-TRDMT1 Flp-in 293 cells were incubated in DMEM with 10% FBS. After overnight incubation with 5 μM 5-azacytidine, the cells were treated with 1 mM H_2_O_2_ for 1 h, then the cells were washed once with PBS and collected in RNA-IP working buffer (50 mM pH 7.5 Tris, 1% NP-40, 0.2% Na-DOC, 0.05% SDS, 1 mM EDTA, 1×Protease inhibitor cocktail, and RNase inhibitor (N2615, Promega; Madison, WI, USA)). The mixtures were pipetted thoroughly and placed on ice for 15 min at 4 °C. Then, the lysates were centrifuged at top speed for 15 min, and the resulting supernatants were incubated with anti-GFP agarose beads. Mixtures were placed on a rotator for 4 h at room temperature. The beads were centrifuged again and washed three times with RNA-IP working buffer. Finally, the beads were mixed with 50 μL 1×RNA fragmentation buffer, heated at 95% for 3 min, and chilled on ice before adding 10 μL reaction stop solution. RNA fragments were purified by ethanol precipitation. For every 20 μL RNA solution, 10 μL 5 M NH_4_OAc and 60 μL pure ethanol were added. The solution was then frozen at −80 °C overnight, centrifuged at top speed for 15 min at 4 °C, and rinsed once with 70% ethanol. The precipitate was dried at room temperature and then dissolved in 10 mM Tris-EDTA (pH 7.5). RNA quantity was measured with a NanoDrop 2000 (ThermoFisher Scientific).

### CRISPR-based LMNA-HR reporter assay

For the assay, a DSB 28 nucleotides upstream to the translational start site of LMNA was created by CRISPR/Cas9, whereas pUC19-LMNA-mClover contains mClover cDNA flanked by 5′ homology and 3′ homology arms of LMNA, which are homologous to sequences upstream and downstream to the break site generated by CRISPR/Cas9 at the LMNA genomic locus. pUC19-LMNA-mClover lacks a mammalian promoter to drive mClover expression and thus does not express mClover fluorescent protein. Cells (WT and TRDMT1 KO) were seeded in a 6-well plate and transfected the next day. Two micrograms of pX459-sgLMNA (expressing Cas9 and a guide RNA targeting LMNA locus), 2 μg of pUC19-LMNA-mClover, and 0.4 μg pCDNA3-mCherry (as a transfection indicator) were transfected using FuGene 6 (Promega). Three days after transfection, cells were analyzed by FACS to assess the number of mClover-positive cells in an mCherry-positive population as described in a previous study^45^. Gating strategy was demonstrated in Supplementary Fig. 8.

### HR/NHEJ flow cytometry

DR-GFP U2OS and Ej5-GFP U2OS stable cells were used for HR and NHEJ assay, respectively. Cells were transfected with the pCMV-I-SceI plasmid. Two days after transfection, the cells were collected for flow cytometry analysis. The normal cell population was gated in P1 by SSC-A and FSC-A. The HR/NHEJ rate was then calculated from the population of GFP-positive cells.

### Cell survival assay

Approximately 400 U2OS cells were seeded in each 6-cm dish and cultured as described above. They were treated with IR 6 h after seeding. After 7–10 days, colonies were fixed and stained with 0.3% crystal violet in methanol, and the number of colonies was counted manually.

### In vitro RNA-protein pull-down assay

3′-biotin-labeled ssRNA (5′-UGACUAAUCGAAGUUGAUACAUCGACGUUA-3′) and complementary ssDNA (5′-TCGTCGTGTTCCTTCGTTGTC-3′) were synthesized from IDT. For m^5^C ssRNA, all five cytidines in the ssRNA sequence were substituted with m^5^C-modified cytidines. The ssRNA with or without m^5^C modification was annealed with ssDNA in a 1:1 ratio.

Purified His-TRDMT1 protein was pulled down with the annealed DNA:RNA hybrid using the Pierce magnetic RNA-Protein pull-down kit (Cat#: 20164, ThermoFisher Scientific) following the manufacturer’s instructions. For in vitro pull-down of RAD52 protein, the wash buffer contained an additional 300 mM NaCl and 0.5 % NP-40. The flow-through and final elution were analyzed by Western blot.

### Protein purification

Rosetta cells harboring pET28b-RAD52 that encode hRAD52 with a C-terminal 6×-His tag were grown to an OD of 0.6 and induced by adding 0.3 mM IPTG for 3 h at 30 °C. Ten grams of overexpressing cells were lysed in lysis buffer (25 mM Tris-HCl [pH 7.5], 500 mM KCl, 1 mM EDTA, 10% glycerol, 1 mM DTT, 0.01% IGEPAL, 1 mM PMSF, and a mixture of protease inhibitors) and sonicated. The lysed sample was centrifuged for 1 h at 40,000×*g*. The cleared supernatant was diluted five times and loaded on Affi-Blue (BIO-RAD) beads in T buffer (25 mM Tris-HCl [pH 7.5], 1 mM EDTA, 10 % glycerol, 1 mM DTT, 0.01% IGEPAL) with 100 mM KCl. Using FPLC, RAD52 was eluted with a gradient of 0–2.5 M of NaSCN in T buffer. Fractions containing RAD52 were pooled together and dialyzed against T buffer with 300 mM KCl and then incubated with Ni-NTA agarose beads for 2 h. RAD52 was then eluted with a gradient of 10–300 mM imidazole in T buffer. Fractions containing RAD52 protein were pooled together and dialyzed as described above, concentrated, and stored at −80 °C for biochemical assays. GST-tagged human TRDMT1 protein was purchased from Sino Biological (Cat#: 11224-H09B). His-tagged human TRDMT1 protein was expressed in Rosetta 2 (Novagen/Millipore) and purified Ni-NTA (Qiagen) and Q-Sepharose (GE Healthcare Life Sciences) columns. *Escherichia coli* Rosetta 2 cells transformed with plasmid expressing His-tagged human TRDMT1 were grown at room temperature overnight in 1 L LB medium with 100 mg ampicillin and 15 mg chloramphenicol. IPTG (0.1 mM) was then added to induce expression, and the culture was incubated for 3 h at 37 °C. Cells were harvested, resuspended in extraction buffer (20 mM HEPES-HCl, 0.3 M NaCl, 20 mM 2ME, 10% glycerol, 20 mM imidazole, and 1% Triton X-100), and sonicated. The cell debris was removed by centrifugation. The cell-free extract was loaded onto Ni-NTA (Qiagen) and Q-Sepharose (GE Healthcare Life Sciences) columns.

### Hybrid substrate preparation

The 5′ ends of oligos were labeled using a 5′ oligonucleotide end-labeling kit (Vector Laboratories; Burlingame, CA, USA) and maleimide-IR800 probe (LI-COR Bioscience; Lincoln, NE, USA). RNA-DNA or m^5^C DNA:RNA hybrid substrates were prepared and conformed similarly to the literature^44^. Briefly, 5′ end-labeled oligo 1 was mixed either with oligo 2 or oligo 3 in buffer H (Tris-HCl [pH 7.5] 90 mM, MgCl_2_ 10 mM, NaCl 50 mM), heat denatured and annealed by slow cooling. Annealed substrates were separated by 10% native PAGE-TAE. The corresponding gel bands were excised and eluted. DNA:RNA or m^5^C DNA:RNA hybrid substrates were confirmed by mobility in native PAGE, heat denaturation, and RNase H treatment^44^.

### Electrophoretic mobility shift assay

5′ maleimide-IR800-labeled substrates were incubated with RAD52 in Buffer B (25 mM Tris-HCl [pH 7.5], 1 mM MgCl_2_, 1 mM DTT, 50 μg/mL BSA) with 50 or 300 mM NaCl and/or 0.5% NP-40 for 15 min at 37 °C. Reactions were loaded on a 6% PAGE-TBE gel and resolved at 4 °C. Gels were imaged using an Odyssey scanner (LI-COR Biosciences). Oligos used in this study are summarized in Supplementary Table 3.

### Mice maintenance and ethics

MDA-MB-231 (6.0 × 10^5^) cells transfected with LV-TRDMT1-RNAi (72262-1; ccAAAGTCATTGCTGCGATAT) or LV-NC-RNAi were injected intraperitoneally into BALB/c nude mice, which were then randomly assigned into one of four groups (three animals per condition). When mice had palpable tumors (~1 week post-injection), the PARPi Olaparib (50 mg/kg, AZD2281; Ku-0059436; Selleck Chemicals, Houston, TX, USA) and/or saline was then intraperitoneally injected into the xenograft tumors once every other day for 20 days. Mice were then killed, and their tumors were harvested. Tumors were fixed, embedded in paraffin, and sectioned into 4-μm-thick slices. After deparaffinization and rehydration, sections were blocked and incubated with antibodies against TRDMT1 (sc-365001, Santa Cruz Biotechnology; Dallas, TX, USA) and Ki-67 (sc-23900, Santa Cruz Biotechnology) and then detected using the Dako Envision two-step method of immunohistochemistry (Carpinteria, CA, USA). All animal experiments were approved by and conducted in accordance with the guidelines established by the Institutional Animal Care and Use Committee at the University of Pittsburgh. Ambient temperature is controlled at 18–26 °C, relative humidity is 40–70%, (12 h light/12 h dark) light change cycle.

### RSI calculations

RSI was calculated using the previously published rank-based linear regression algorithm^46^. Higher RSI indicates possible radioresistance compared to lower RSI. *RSI* = − 0.0098009 * AR + 0.0128283 * cJun + 0.0254552 * STAT1 − 0.0017589 * PKC − 0.0038171 * RelA + 0.1070213 * cABL − 0.0002509 * SUMO1 − 0.0092431 * PAK2 − 0.0204469 * HDAC − 0.0441683 * IRF1. As previously described, the median value of TRDMT1 and BRCA1 TPM was defined as the cutoff value to classify patients into high expression and low expression groups.

### Reporting summary

Further information on research design is available in the Nature Research Reporting Summary linked to this Article.

## Supplementary information

Supplementary Information

Reporting Summary

## Data Availability

The source data underlying Figs. 1a, b, d–f, 2a, c, d–f, 3a–c, d–f, 4b, d, f, g, 5a–d and Supplementary Figs. 1c–e, 2a, c, e, 3a, b, e, 4a–c, 5b–h, 6a–d, f, 7c are provided in the Source Data file including uncropped gels, blots, and all reported averages in graphs. All data are available from the authors upon reasonable request.

## Source data

Source Data
