# Supplementary material for: m5C modification of mRNA serves a DNA damage code to promote homologous recombination
Source: Nat Commun. 2020 Jun 5;11:2834. doi: 10.1038/s41467-020-16722-7 (PMC7275041; doi:10.1038/s41467-020-16722-7)
Supplement: Supplementary file 1 — Supplementary Information [file 41467_2020_16722_MOESM1_ESM.pdf]

Supplementary Information

m^5C Modification of mRNA Serves a DNA Damage Code to Promote Homologous Recombination

Chen et al.

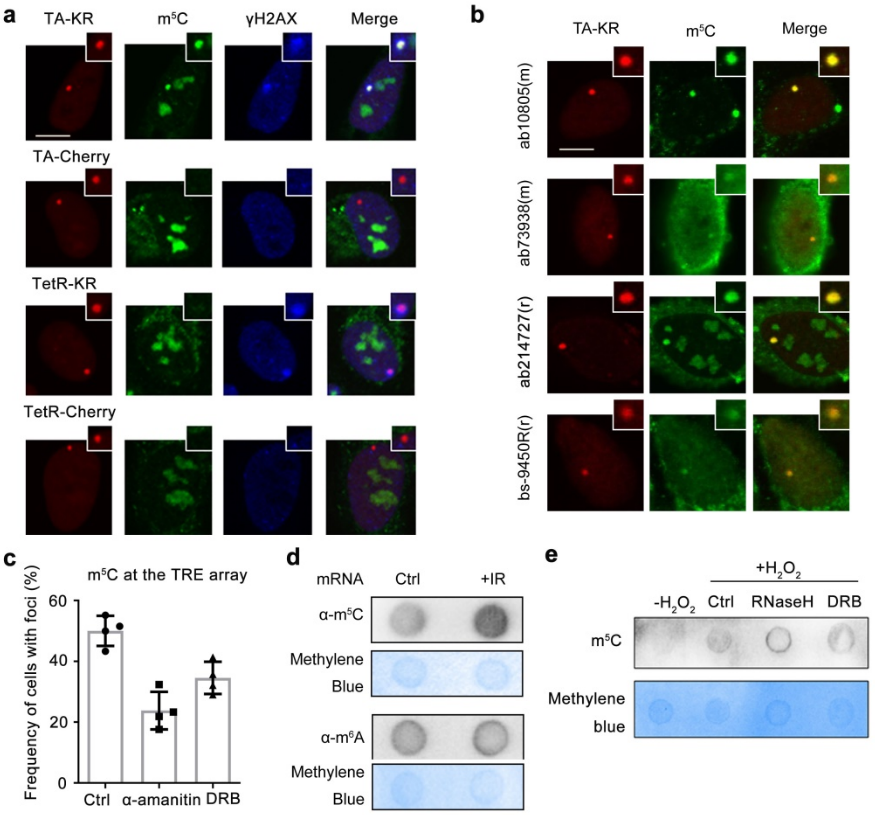

Supplementary Figure 1. m^{5}C mRNA methylation at damage sites is dependent on active transcription. a. U2OS-TRE cells transfected with TA-KR/TA-Cherry/tetR-KR/tetR-Cherry plasmids were exposed to light for 30 min for KR activation and allowed to recover for 1 h before harvest. The cells were stained with m^{5}C and γH2AX antibody (scale bar: 10 μm). **b**. U2OS-TRE cells transfected with TA-KR to induce local oxidative damage or for a control. Representative images for m^{5}C staining with four different anti-m^{5}C antibodies were shown (scale bar: 10 μm). **c**. U2OS-TRE cells transfected with TA-KR were treated with 20 μM DRB (24 h) or 100 μg/ml α-amanitin (2 h) before damage and stained for m^{5}C (n=4, Mean± SEM, 50 cells per replicate). **d**. U2OS cells were treated with 4 Gy IR with 3.5 h of recovery. The mRNA was then extracted from the cell lysate and analyzed via dot blot with m^{5}C and m^{6}A antibodies. **e**. U2OS cells with RNase H1 overexpression or 20 μM DRB (24 h) treatment were treated with 2 mM H_2O_2 for 40 min before mRNA extraction for m^{5}C dot blot analysis.

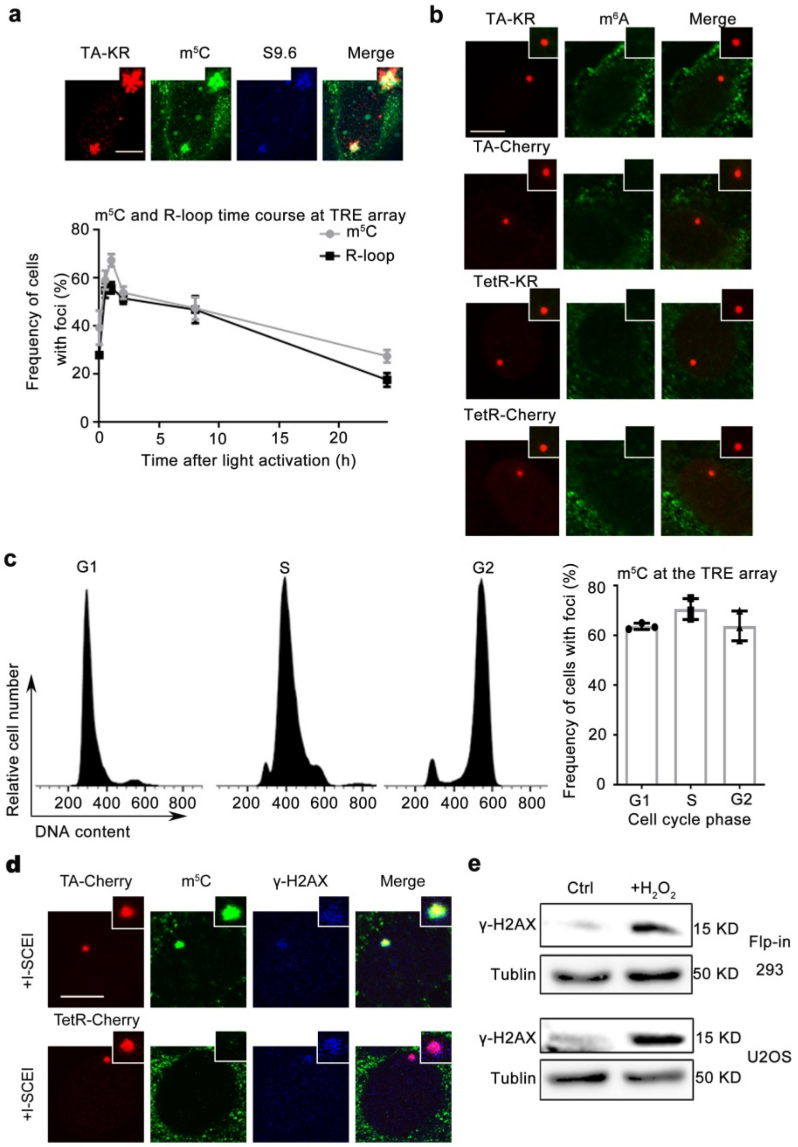

Supplementary Figure 2. m^5C mRNA methylation is enriched at transcriptionally active sites with DNA damage. a. U2OS-TRE cells transfected with TA-KR were exposed to light for 30 min for KR activation allowed to recover for different time before harvest. The cells were stained with m^5C and S9.6 antibody (For each time point, n=3, Mean ± SEM, 50 cells per replicate). **b.** U2OS-TRE cells were transfected with TA-KR or TA-Cherry. The cells were stained with m^6A antibody right after light activation. **c.** U2OS-TRE cells transfected with TA-KR were synchronized to different phase using double thymidine block method. The cells were exposed to light for 30 min for KR activation and allowed to recover for 1 h before harvest. The cells were stained with m^5C antibody (n=3, Mean ± SD, 50 cells per replicate). **d.** U2OS-TRE cells were transfected with I-SceI and TA-Cherry/tetR-Cherry to trigger pure DSBs. The cells were than stained for m^5C and γH2AX. **e**. Flp-in 293 or U2OS cells were treated with 2 mM H_2O_2 for 40 min before harvested for WB analysis, respectively. For a, b and d, scale bar: 10 µm.

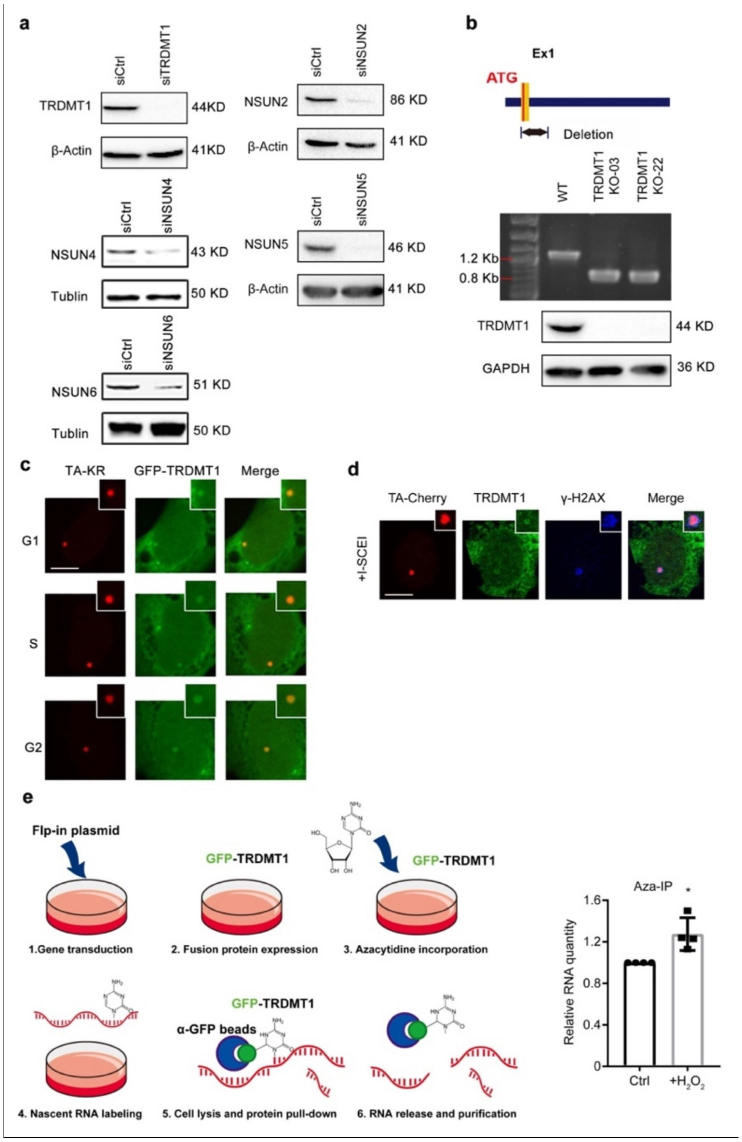

Supplementary Figure 3. TRDMT1 mediates the m^{5}C mRNA methylation in the context of DNA:RNA hybrids. a. Methyltransferases in U2OS-TRE cells pre-treated with control or the indicated siRNA were measured by Western blot. **b.** TRDMT1-KO U2OS-TRE cells were generated with CRISPR-Cas9 and confirmed by genomic PCR and Western blot. **c**. U2OS-TRE cells co-transfected with TA-KR, GFP-TRDMT1 were synchronized to different phase as in Figure S2c. The cells were exposed to light for 30 min for KR activation and allowed to recover for 1 h before harvest (scale bar: 10 μm). **d.** U2OS-TRE cells were co-transfected with I-SceI, TA-Cherry and GFP-TRDMT1 to trigger pure DSBs. The cells were then stained with γH2AX (scale bar: 10 μm).**e.** Flp-in 293 cells stably expressing GFP-TRDMT1 were pre-incubated with 5 μM 5-azacytidine overnight. The cells were then incubated in 1 mM H_2O_2 for 1 h. GFP-TRDMT1 was pulled down by anti-GFP magnetic beads, and the bound RNA was purified and measured (n=4, Mean ± SD). Statistical analysis was performed with the unpaired two tailed Student's *t* test. *$p < 0.05$.

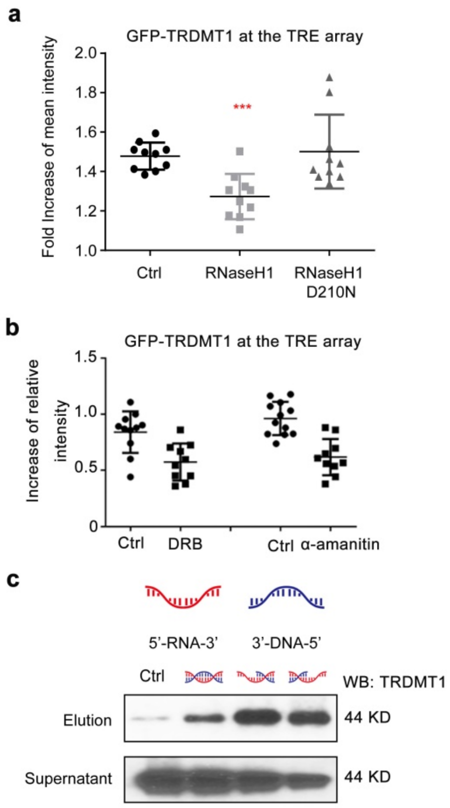

Supplementary Figure 4. TRDMT1 responds to DNA damage in the context of DNA:RNA hybrids. a. U2OS-TRE cells co-transfected with TA-KR, GFP-TRDMT1, and HA-RNase H1-WT/HA-RNase H1-D210N were irradiated by light and collected (n = 10, Mean ± SD). **b**. U2OS-TRE cells co-transfected with TA-KR, GFP-TRDMT1 were treated with 20 μM DRB (24 h) or 100 μg/ml α-amanitin (2 h) before damage. The cells were irradiated by light and collected (n = 10, Mean ± SD). **c**. TRDMT1 protein pull down by biotin-labeled DNA:RNA hybrids with blunt-end or 3'/5' ssRNA overhangs. Statistical analysis was performed with the unpaired two tailed Student's *t* test. ***p < 0.001.

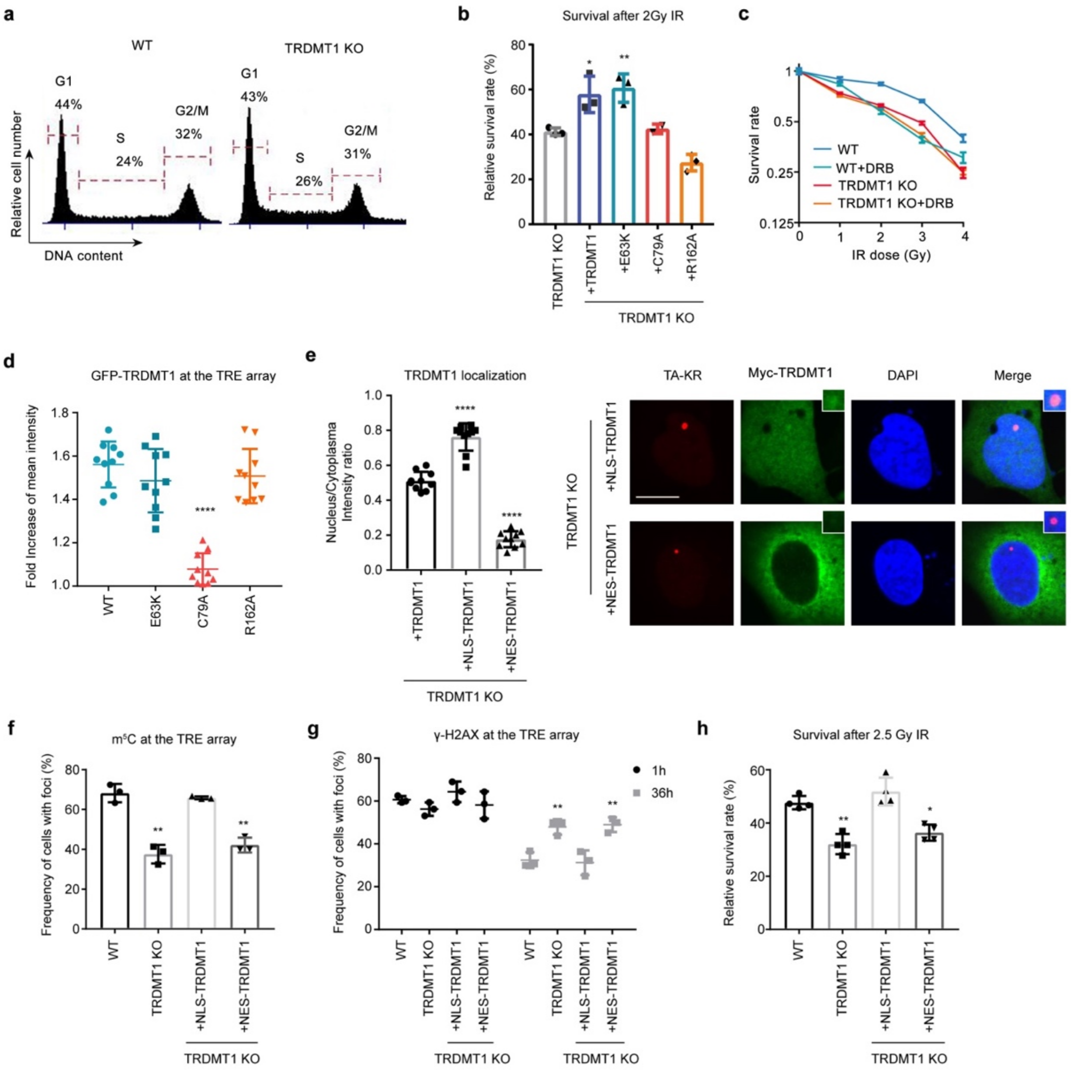

Supplementary Figure 5. Nuclear TRDMT1 is essential for regulating DNA damage repair. **a.** WT or TRDMT1 KO U2OS-TRE cell lines were stained with PI after RNA digestion and applied to flow cytometry analysis. **b.** U2OS-TRE WT, TRDMT1 KO, and TRDMT1 stably expressing cells were treated with 2 Gy IR to measure the survival rate via a colony formation assay (n=3, Mean ± SD). **c.** The WT or TRDMT1 KO U2OS-TRE cell treated with 20 μM DRB (24 h) were exposed to ionizing radiation (IR). The survival rate was measured via colony formation assay (n = 3, Mean ± SD). **d.** U2OS-TRE cells co-transfected with TA-KR and GFP-TRDMT1 (WT or E63K, C79A, and R162A mutants) were harvested after damage (n=10, Mean ± SD). **e.** TRDMT1 KO U2OS-TRE cells were infected by LV for the stable expression of NLS- and NES-TRDMT1. The stable cell lines were transfected with TA-KR and then stained with anti-Myc antibody and DAPI. The TRDMT1 expression ratio in the nucleus/cytoplasm is calculated (scale bar: 10 μm) (n=10, Mean ± SD). **f-g.** WT, TRDMT1-KO, and the two rescued lines were transfected with TA-KR and harvested at the indicated time points to stain for m^5C or γH2AX (n=3, Mean ± SD). **h.** The four cell lines were irradiated with 0 or 2.5 Gy IR. The relative survival rate was counted via a colony formation assay (n=4, Mean ± SD). Statistical analysis was performed with the unpaired two tailed Student's *t* test. *p < 0.05; **p < 0.01; ***p < 0.001; ****p < 0.0001.

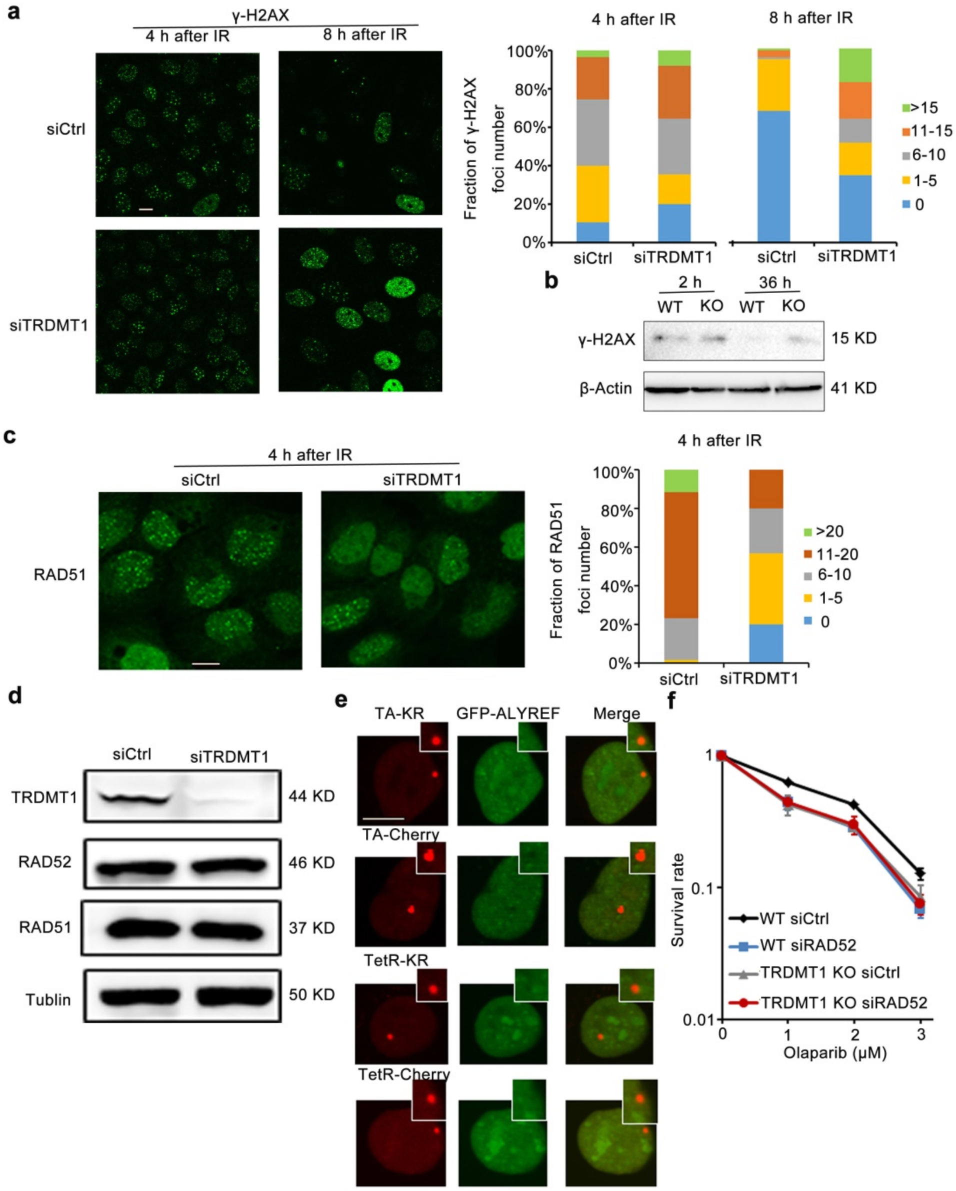

Supplementary Figure 6. TRDMT1 contributes to homologous recombination. a-b. U2OS-TRE cells pretreated with TRDMT1 siRNA were exposed to 8 Gy IR irradiation and allowed to recover for different time. The number of γ-H2AX foci per cell was quantified (a). The γ-H2AX quantity was measured by western blot (b). **c**. U2OS-TRE cells pretreated with TRDMT1 siRNA were exposed to 6 Gy IR irradiation and allowed to recover for 4 h. The number of RAD51 foci per cell was quantified. **d**. U2OS-TRE cells pretreated with TRDMT1 siRNA were collected for western blot anlysis. **e**. U2OS-TRE cells transfected with TA-KR/TA-Cherry/tetRKR/tetR-Cherry and GFP-ALYREF plasmids were were exposed to light for 30 min and allowed to recover for 1 h before harvest. **f**. WT and TRDMT1 KO U2OS cells pretreated with control siRNA or RAD52 siRNA were treated with Olaparib at the indicated dose. The survival rate was measured via the colony formation assay (n=3, Mean ± SEM). For a, c and e, scale bar: 10 µm.

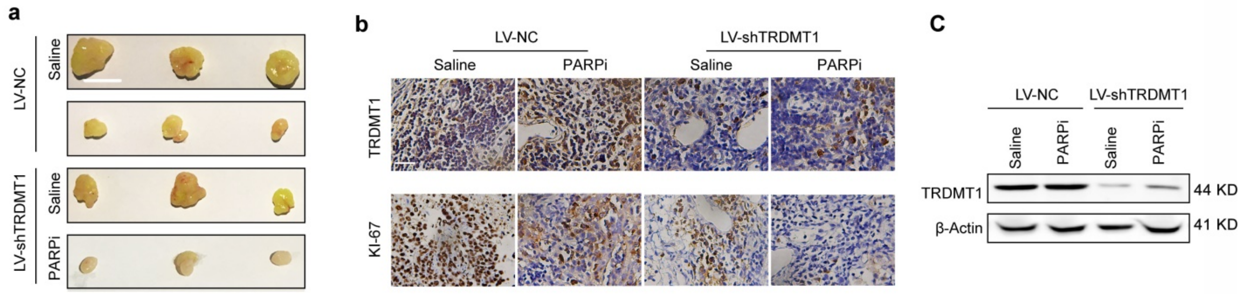

Supplementary Figure 7. TRDMT1 suppression increases cell sensitivity to PARPi for inhibiting tumor growth. a. Representative images of tumors in a xenograft transfected with LV-shTRDMT1/LV-NC and treated with saline or Olaparib. **b.** IHC staining of fixed tumors stained with TRDMT1 and Ki-67 (scale bar: 50 μm). **c**. TRDMT1 western blot analysis was performed for the tumor samples.

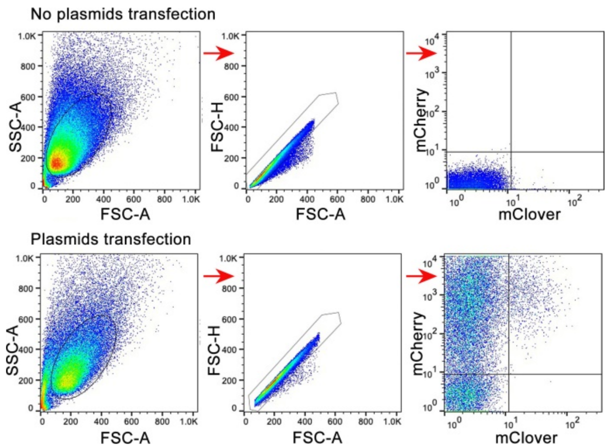

Supplementary Figure 8. | Gating strategy used in flow cytometry analysis of CRISPR-based LMNA-HR Reporter Assay. U2OS WT cells with or without plasmids transfection were analyzed.

Supplementary Table 1.

Antibody	Species	Clone, Catalog no.	Company
TRDMT1 (DNMT2)	Mouse monoclonal	D-9, sc-365001	Santa Cruz Biotechnology
5-methylcytosine (m^5C)	Mouse monoclonal	33D3, ab10805	Abcam
5-methylcytosine (m^5C)	Rabbit monoclonal	RM231, ab214727	Abcam
5-methylcytosine (m^5C)	Mouse monoclonal	5MC-CD, ab73938	Abcam
5-methylcytosine (m^5C)	Rabbit polyclonal	bs-9450R	Bioss Antibodies
N6-methyladenosine (m^6A)	Mouse monoclonal	17-3-4-1, MABE1006	EMD Millipore
PCNA (Ab-1)	Mouse monoclonal	PC10, NA03	EMD Millipore
NSUN5	Mouse monoclonal	H-10, sc-376147	Santa Cruz Biotechnology
NSUN2	Rabbit polyclonal	20854-1-AP	Proteintech
NSUN4	Rabbit polyclonal	PA5-55876	Invitrogen
NSUN6	Mouse monoclonal	D5, sc393446	Santa Cruz Biotechnology
RAD51	Rabbit polyclonal	ab63801	Abcam
S9.6	Mouse monoclonal	ENH001	Kerafast
GFP	Mouse monoclonal	11814460001	Roche
HA-tag	Rabbit polyclonal	ab9110	Abcam
Myc-tag	Mouse monoclonal	9E10, ab32	Abcam
Flag	Mouse monoclonal	M2, IB13026	Eastman Kodak
RAD52	Mouse monoclonal	F-7, sc-365341	Santa Cruz Biotechnology
ɣH2AX, ser139	Mouse monoclonal	JBW301, 05–636	EMD Millipore
KI-67	Mouse monoclonal	sc-23900	Santa Cruz Biotechnology
β-Actin	Mouse monoclonal	8H10D10	Cell Signaling Technology

Supplementary Table 2. Primers used to generate TRDMT1 KO cells and TRDMT1 mutant plasmids

CRISPR-Cas 9 TRDMT1 KO upstream sgRNA	DNMT2 UP B1	CACCGACCGGCAGGCCTAGCTCCG
	DNMT2 UP B2	AAACCGGAGCTAGGCCTGCCGGTC
CRISPR-Cas 9 TRDMT1 KO downstream sgRNA	DNMT2 DOWN A1	CACCGTTGGGAGTCGGGATTCGCA
	DNMT2 DOWN A2	AAACTGCGAATCCCGACTCCCAAC
TRDMT1 E63K mutant	E63KF	ATTGAAGGCATTACACTCAAAGAGTTTGACAGATTATCTT
	E63KR	AAGATAATCTGTCAAACTCTTTGAGTGTAATGCCTTCAAT
TRDMT1 C79A mutant	C79AF2	GAGCCCTCCCGCCCAGCCATTCACAAGGATTGGCCG
	C79AR2	GAATGGCTGGGCGGGAGGGCTCATTAAAATCATATC
TRDMT1 R162A mutant	R162AF	GGCATTCCAAATTCAAGGCTAGCATATTTTCTTATTGCAAAG
	R162AR	CTTTGCAATAAGAAAATATGCTAGCCTTGAATTTGGAATGCC

Supplementary Table 3.

S. No	Oligo name	Sequence
1	Oligo 1 (ssDNA 50 nts)	ATCATCACCATAACGTCGATGTATCAACTTCGATTAGTCA CACCAATTAA
2	Oligo 2 (ssRNA 50 nts)	UUAAUUGGUGUGACUAAUCGAAGUUGAUACAUCGACGU UAUGGUGAUGAU
3	Oligo 3 (5mcRNA 50 nts)*	UUAAUUGGUGUGACUAAUCGAAGUUGAUACAUCGACGU UAUGGUGAUGAU

* Oligo 3 is methylated at each C.
