# Supplementary material for: m5C modification of mRNA serves a DNA damage code to promote homologous recombination
Source: Nat Commun. 2020 Jun 5;11:2834. doi: 10.1038/s41467-020-16722-7 (PMC7275041; doi:10.1038/s41467-020-16722-7)
Supplement: Supplementary file 2 — Reporting Summary [file 41467_2020_16722_MOESM2_ESM.pdf]

nature research

Corresponding author(s): NCOMMS-19-40462A

Last updated by author(s): May 9, 2020

Reporting Summary

Nature Research wishes to improve the reproducibility of the work that we publish. This form provides structure for consistency and transparency in reporting. For further information on Nature Research policies, see Authors & Referees and the Editorial Policy Checklist.

Statistics

For all statistical analyses, confirm that the following items are present in the figure legend, table legend, main text, or Methods section.

n/a	Confirmed	
☐	☒	The exact sample size (*n*) for each experimental group/condition, given as a discrete number and unit of measurement
☐	☒	A statement on whether measurements were taken from distinct samples or whether the same sample was measured repeatedly
☐	☒	The statistical test(s) used AND whether they are one- or two-sided<br>*Only common tests should be described solely by name; describe more complex techniques in the Methods section.*
☒	☐	A description of all covariates tested
☒	☐	A description of any assumptions or corrections, such as tests of normality and adjustment for multiple comparisons
☐	☒	A full description of the statistical parameters including central tendency (e.g. means) or other basic estimates (e.g. regression coefficient) AND variation (e.g. standard deviation) or associated estimates of uncertainty (e.g. confidence intervals)
☐	☒	For null hypothesis testing, the test statistic (e.g. *F*, *t*, *r*) with confidence intervals, effect sizes, degrees of freedom and *P* value noted<br>*Give P values as exact values whenever suitable.*
☒	☐	For Bayesian analysis, information on the choice of priors and Markov chain Monte Carlo settings
☒	☐	For hierarchical and complex designs, identification of the appropriate level for tests and full reporting of outcomes
☒	☐	Estimates of effect sizes (e.g. Cohen's *d*, Pearson's *r*), indicating how they were calculated

Our web collection on statistics for biologists contains articles on many of the points above.

Software and code

Policy information about availability of computer code

Data collection	FV1000 confocal software 4.2, Image lab 5.2
Data analysis	ImageJ 1.51k, Graphpad prism 6.01, Flowjo 10.6.2

For manuscripts utilizing custom algorithms or software that are central to the research but not yet described in published literature, software must be made available to editors/reviewers. We strongly encourage code deposition in a community repository (e.g. GitHub). See the Nature Research guidelines for submitting code & software for further information.

Data

Policy information about availability of data

All manuscripts must include a data availability statement. This statement should provide the following information, where applicable:

- Accession codes, unique identifiers, or web links for publicly available datasets
- A list of figures that have associated raw data
- A description of any restrictions on data availability

The source data underlying Figs. 1a, b, d, e, f, 2a, c, d, e, f, 3a, b, c, d, e, f, 4b, d, f, g, 5b, c, d, Supplementary Figs. 1c, d, e, 2a, c, e, 3a, b, e, 4a, b, c, 5b–h, 6a, b, c, d, f, 7c are provided in the Source Data File including uncropped gels, blots and all reported averages in graphs

Field-specific reporting

Please select the one below that is the best fit for your research. If you are not sure, read the appropriate sections before making your selection.

☒ Life sciences ☐ Behavioural & social sciences ☐ Ecological, evolutionary & environmental sciences

Life sciences study design

All studies must disclose on these points even when the disclosure is negative.

Sample size	Sample size, number of replicates, error bars and statistical tests were chosen based on accepted practices in the field and stated in each figure legend. Generally, experiments were performed independently and reproduced using at least three biological replicates.
Data exclusions	no data were excluded from analysis
Replication	Each experiment was repeated multiple times with similar results. All attempts at replicating data were successful. In experiments where variation is inherent to the assay, data from independent experiments were pooled
Randomization	Animals were assigned randomly. Other experiments, not applicable
Blinding	The investigators were not blinded during data collection.

Reporting for specific materials, systems and methods

We require information from authors about some types of materials, experimental systems and methods used in many studies. Here, indicate whether each material, system or method listed is relevant to your study. If you are not sure if a list item applies to your research, read the appropriate section before selecting a response.

Materials & experimental systems

n/a	Involved in the study	
☐	☒	Antibodies
☐	☒	Eukaryotic cell lines
☒	☐	Palaeontology
☐	☒	Animals and other organisms
☒	☐	Human research participants
☒	☐	Clinical data

Methods

n/a	Involved in the study	
☒	☐	ChIP-seq
☐	☒	Flow cytometry
☒	☐	MRI-based neuroimaging

Antibodies

Antibodies used

Antibody Species Clone, Catalog no. Company
TRDMT1 (DNMT2) Mouse monoclonal D-9, sc-365001 Santa Cruz Biotechnology
5-methylcytosine (m5C) Mouse monoclonal 33D3, ab10805 Abcam
5-methylcytosine (m5C) Rabbit monoclonal RM231, ab214727 Abcam
5-methylcytosine (m5C) Mouse monoclonal 5MC-CD, ab73938 Abcam
5-methylcytosine (m5C) Rabbit polyclonal bs-9450R Bioss Antibodies
N6-methyladenosine (m6A) Mouse monoclonal 17-3-4-1, MABE1006 EMD Millipore
PCNA (Ab-1) Mouse monoclonal PC10, NA03 EMD Millipore
NSUN5 Mouse monoclonal H-10, sc-376147 Santa Cruz Biotechnology
NSUN2 Rabbit polyclonal 20854-1-AP Proteintech
NSUN4 Rabbit polyclonal PA5-55876 Invitrogen
NSUN6 Mouse monoclonal D5, sc393446 Santa Cruz Biotechnology
RAD51 Rabbit polyclonal ab63801 Abcam
S9.6 Mouse monoclonal ENH001 Kerafast
GFP Mouse monoclonal 11814460001 Roche
HA-tag Rabbit polyclonal ab9110 Abcam
Myc-tag Mouse monoclonal 9E10, ab32 Abcam
Flag Mouse monoclonal M2, IB13026 Eastman Kodak
RAD52 Mouse monoclonal F-7, sc-365341 Santa Cruz Biotechnology
ɣH2AX, ser139 Mouse monoclonal JBW301, 05–636 EMD Millipore
KI-67 Mouse monoclonal sc-23900 Santa Cruz Biotechnology
β-Actin Mouse monoclonal 8H10D10 Cell Signaling Technology

Validation

All antibodies are commercially available and have been validated by suppliers and previous publications. Antibodies were used according to manufacture instructions

Eukaryotic cell lines

Policy information about cell lines

Cell line source(s)	U2OS TRE cell, Flp-in 293, 293FT, MDA-MB-231
Authentication	Cell lines were not authenticated. U2OS cells were purchased from ATCC and integrated with TRE repeats. Flp-in 293 cell line was purchased from Thermo Fisher.
Mycoplasma contamination	No Mycoplasma contamination of cell lines. BM-cyclin (Sigma-Aldrich Cat 1079905001) was used during culture and removed before experiments. Mycoplasma PCR ELISA (Roche 11663925910) was used.
Commonly misidentified lines (See ICLAC register)	no commonly misidentified line were used

Animals and other organisms

Policy information about studies involving animals; ARRIVE guidelines recommended for reporting animal research

Laboratory animals	Species and Strain: BALB/c nude mice, Sex: Female, Age:4-5 weeks, Weight:20g
Wild animals	no wild animals were used
Field-collected samples	no filed-collected samples
Ethics oversight	All animal experiments were approved by and conducted in accordance with the guidelines established by the Institutional Animal Care and Use Committee at the University of Pittsburgh

Note that full information on the approval of the study protocol must also be provided in the manuscript.

Flow Cytometry

Plots

Confirm that:

☒ The axis labels state the marker and fluorochrome used (e.g. CD4-FITC).

☒ The axis scales are clearly visible. Include numbers along axes only for bottom left plot of group (a 'group' is an analysis of identical markers).

☒ All plots are contour plots with outliers or pseudocolor plots.

☒ A numerical value for number of cells or percentage (with statistics) is provided.

Methodology

Sample preparation	the sample preparation has been described in Materials and Methods section
Instrument	LSR II
Software	flowjo 10.6.2
Cell population abundance	at least 10,000 cells were collected for each experiment
Gating strategy	gating strategy was described in methods

☒ Tick this box to confirm that a figure exemplifying the gating strategy is provided in the Supplementary Information.
